# *Satureja khuzistanica* Jamzad essential oil and pure carvacrol attenuate TBI-induced inflammation and apoptosis via NF-*κ*B and caspase-3 regulation in the male rat brain

**DOI:** 10.1038/s41598-023-31891-3

**Published:** 2023-03-23

**Authors:** Elham Abbasloo, Sedigheh Amiresmaili, Sara Shirazpour, Mohammad Khaksari, Firas Kobeissy, Theresa Currier Thomas

**Affiliations:** 1 Endocrinology and Metabolism Research Center, Institute of Basic and Clinical Physiology Sciences, Kerman University of Medical Sciences, Kerman, Iran; 2 Department of Physiology, Bam University of Medical Sciences, Bam, Iran; 3 Department of Physiology and Pharmacology, Faculty of Medicine, Kerman University of Medical Science, Kerman, Iran; 4 Physiology Research Center, Institute of Neuropharmacology, Kerman University of Medical Sciences, Kerman, Iran; 5 Department of Biochemistry and Molecular Genetics, Faculty of Medicine, American University of Beirut, Beirut, Lebanon; 6 Department of Child Health, University of Arizona College of Medicine - Phoenix, Phoenix, USA; 7 Translational Neurotrauma Research Program, Barrow Neurological Institute at Phoenix Children’s Hospital, Phoenix, USA; 8 Center for Neurotrauma, Multiomics and Biomarkers, Morehouse School of Medicine, Atlanta, Georgia USA

**Keywords:** Neuroscience, Physiology

## Abstract

Traumatic brain injury (TBI) causes progressive dysfunction that induces biochemical and metabolic changes that lead to cell death. Nevertheless, there is no definitive FDA-approved therapy for TBI treatment. Our previous immunohistochemical results indicated that the cost-effective natural Iranian medicine, *Satureja khuzistanica* Jamzad essential oil (SKEO), which consists of 94.16% carvacrol (CAR), has beneficial effects such as reducing neuronal death and inflammatory markers, as well as activating astrocytes and improving neurological outcomes. However, the molecular mechanisms of these neuroprotective effects have not yet been elucidated. This study investigated the possible mechanisms involved in the anti-inflammatory and anti-apoptotic properties of SKEO and CAR after TBI induction. Eighty-four male Wistar rats were randomly divided into six groups: Sham, TBI, TBI + Vehicle, TBI + CAR (100 and 200 mg/kg), and TBI + SKEO (200 mg/kg) groups. After establishing the “Marmarou” weight drop model, diffuse TBI was induced in the rat brain. Thirty minutes after TBI induction, SKEO & CAR were intraperitoneally injected. One day after TBI, injured rats exhibited significant brain edema, neurobehavioral dysfunctions, and neuronal apoptosis. Western blot results revealed upregulation of the levels of cleaved caspase-3, NFκB p65, and Bax/Bcl-2 ratio, which was attenuated by CAR and SKEO (200 mg/kg). Furthermore, the ELISA results showed that CAR treatment markedly prevents the overproduction of the brain pro-inflammatory cytokines, including IL-1*β*, TNF-α, and IL-6. Moreover, the neuron-specific enolase (NSE) immunohistochemistry results revealed the protective effect of CAR and SKEO on post-TBI neuronal death. The current study revealed that the possible neuroprotective mechanisms of SKEO and CAR might be related to (at least in part) modulating NF-κB regulated inflammation and caspase-3 protein expression. It also suggested that CAR exerts more potent protective effects than SKEO against TBI. Nevertheless, the administration of SKEO and CAR may express a novel therapeutic approach to ameliorate TBI-related secondary phase neuropathological outcomes.

## Introduction

It is estimated that 69 million traumatic brain injuries (TBIs) occur each year globally which impose a significant therapeutic burden on human societies. Traffic accidents and TBI are the third most common causes of death worldwide, according to data provided by the World Health Organization^1,2^. The incidence of TBI is highest among high-income countries, while the burden is greatest in low-income and middle-income countries^3^. Therefore, cost-effective treatments for TBI are still important^4^.

It has been well established that TBI's highly complex pathophysiological process involves primary disruption of brain tissue due to direct mechanical trauma and secondary injury. While the primary phase occurs at the injury site within minutes to hours post-injury, the secondary injury occurs hours to days later. The secondary injury involves a series of neuropathological events, including blood–brain barrier (BBB) disruption, inflammation, excitotoxic damage, mitochondrial dysfunction, oxidative stress, lipid peroxidation, necrotic, and apoptotic cell death^5^. Among these events, persistent and excessive inflammation can worsen the neurological disruption during the secondary insult process by secretion of pro-inflammatory mediators, such as tumor necrosis factor-α (TNF-α), which plays an essential role in releasing interleukin-6 (IL-6) and interleukin-1β (IL-1β) by T-cells^6^. Increased IL-6 production causes neuronal impairment, BBB damage, and other acute neurological complications^7^. Many experimental data suggested that nuclear factor kappa B (NF-κB) activation enhances the transcription of pro-inflammatory cytokines^8^, and the cytokines are known to, in turn, activate NF-κB^9^. In this regard, the pro-apoptotic family members, such as B-cell lymphoma protein 2 (Bcl-2)-associated (Bax), triggering cytokine release, leading to caspase family activation^10–12^.

Our previous study findings showed that *Satureja khuzistanica* Jamzad essential oil (SKEO) with a high percentage of carvacrol (CAR) exerts a neuroprotective effect against the TBI pathophysiology in rats^13^. SKEO can reduce BBB permeability and edema, regulating astrocytes, neurons, and blood origin-infiltrated cells that produce cytokines, and decrease neuronal death followed by improved neurological function^13^. However, the protective mechanisms of SKEO against TBI complications are still unknown.

*Satureja khuzistanica* Jamzad (SKJ) (also known as Marzeh Khuzestan in Persian) is a member of the *Satureja* genus, which belongs to the Lamiaceae family and the Nepetoidae subfamily. It is found in the southwestern and southern sections of Iran. This plant is utilized as a dental anesthetic drop and mouth disinfectant in traditional medicine, as well as in the food and pharmaceutical industries^14,15^. This plant contains more than 4.5% essential oil^14^, and CAR is the most abundant compound in SKEO (90.08–94.16%) (see Table 1)^16,17^. Accumulating evidence has reported anti-nociceptive^18^, antioxidant^19^, anti-allergic, anti-apoptotic, neuroprotective, and anti-inflammatory effects of this plant essential oil and extract^20–22^.

**Table 1:** Composition of *Satureja khuzistanica* Jamzad essential oil.

Rt	RI	KI	Compound	Area%
3.971	846.9895	847	Ethyl 2-methylbutanoate	0.07
5.405	928.0449	929	α-Thujene	0.37
5.613	937.3933	937	α-Pinene	0.26
6.694	985.9775	986	Beta-pinene	0.09
6.802	990.8315	990	Myrcene	0.7
7.363	1013.222	1013	α-Phellanderene	0.05
7.619	1022.704	1023	α-Terpinene	0.12
7.879	1032.333	1034	P-Cymene	2.29
7.955	1035.148	1035	Limonene	0.08
8.045	1038.481	1037	β-Phellandrene	0.06
8.728	1063.778	1063	γ-Terpinene	0.27
9.179	1080.481	1085	Cis sabinene hydrate	0.42
9.89	1106.626	1106	l-linalool	0.68
10.091	1113.864	1116	Trans sabinene hydrate	0.12
12.282	1192.762	1193	Endo-borneol	0.14
12.424	1197.875	1200	4-Terpineol	0.55
12.878	1213.251	1217	α-Terpinol	0.08
12.934	1248.675	1244	Iso thymol methyl ether	0.14
15.692	1308.1691	1308	Thyme camphor	0.3
**16.253**	**1328.2694**	**1327**	**Carvacrol**	**91.33**
19.098	1431.8113	1431	Caryophyllene	0.18
21.25	1513.6526	1510	Beta-Bisabolene	0.58
23.512	1603.3571	1606	Caryophyllene oxide	0.17

RI^1^; Retention indices determined relative to n-alkanes (C_6_–C_24_) on a DB-5GC column.

RI: Retention indices; MS: mass spectra; Col: co-injection.

Significant values are in bold.

Carvacrol (2-methyl-5-(1-methyl ethyl)-phenol) is found in the genus of plants belonging to the Lamiaceae family, such as *Thym*, *Satureja*, and *Origanum,* so oils derived from these plants can contain 85–90% carvacrol^23,24^. Because of its low molecular mass and lipophilic characteristics, this molecule can easily cross the BBB^25^. CAR possesses various biological and pharmacological properties in vitro and in vivo, including antioxidant, anticancer, antibacterial, antifungal, anti-inflammatory, and hepatoprotective effects^26–28^. Previous studies showed that CAR provided neuroprotection against ischemia reperfusion-induced cerebral injury^29^, spinal cord injury^30^, and neurodegenerative disease^31^. In 2012 Peter and colleagues declared that inhibition of transient receptor potential channel (TRPC1) by CAR enhanced neurological recovery after a TBI in mice^32^. However, the underlying mechanisms of these neuroprotective processes have yet to be fully clarified in TBI.

Based on the previously mentioned evidence about the protective activity of SKEO and CAR, the current study evaluates the potential neuroprotective mechanisms of CAR and SKEO post-TBI induction. In this regard, we investigated whether CAR and SKEO could regulate apoptosis-related proteins; caspase-3, Bax, and Bcl2, as well as the pro-inflammatory regulatory cytokine, NF-κB, to ameliorate the neurological deficits observed during the acute phase of TBI.

## Materials and methods

### Animals and experimental protocol

Male Wistar rats (200–250 g) were maintained in a temperature-controlled room (22–25 °C) with a regular 12-h light/dark cycle and free access to food and water. Rats were randomly divided into six groups, as follows: (1) Sham: these rats underwent all preliminary procedures for TBI except for the TBI induction (weight dropped), (2) TBI: these rats were exposed to the brain trauma and received no treatment, (3) TBI + Veh: these rats intraperitoneally injected with vehicle (tween 20, 1% i.p)^33,34^, (4) TBI + CAR100: these rats received CAR (100 mg/kg, i.p), (5) TBI + CAR200: these rats received CAR (200 mg/kg, i.p), (6) TBI + SKEO200: these rats received SKEO (200 mg/kg, i.p). All treatments and vehicles were administered 30 min after TBI induction. To achieve 90% power- to detect statistical significance at 95% confidence interval, six rats were assigned to each group. The rats were sacrificed 24 h after the TBI^35^ (Table 1 and Supplemental Fig. 1).

A dose of 100 mg/kg of CAR was shown to be ineffective based on data obtained from the veterinary coma scale (VCS) and brain water content (BWC), but a dose of 200 mg/kg of CAR was associated with improved VCS and reduced BWC. As a result, the following experiments were carried out using a 200 mg/kg CAR and SKEO. In this regard, our previous dose–response analysis revealed that the SKEO (200 mg/kg) is the most beneficial dose^13^. Amanlou et al. found that intraperitoneal treatment of dosages greater than 200 mg/kg of SKJ was related to sleepiness and decreased physical activity^19^. In a pilot study, we observed that CAR (250 mg/kg) caused drowsiness (somnolent-like behavior) and staggering in rats by a similar mechanism to SKEO (250 mg/kg). As a result, we avoided utilizing higher doses due to the potential risk of interfering with neurological consciousness-dependent outcomes (e.g., VCS).

Furthermore, vehicle administration (Tween^®^ 20, 1%) caused no significant difference between the TBI and TBI + Veh groups in the water content, VCS, and ELISA tests; hence, we only examined the indicated proteins expression and neural death in the TBI + Veh group. As a result, the number of utilized animals and associated research costs were reduced.

All experiments were performed in accordance with relevant guidelines and regulations and adhered to the ARRIVE guidelines (https://arriveguidelines.org/) which was used for reporting of animal experiments. The study was reviewed and approved by the local ethical committee of the Kerman University of Medical Sciences (Ethics code No. 1395.703).

### Chemicals and essential oil preparation

Tween^®^ 20 and CAR were purchased from Merck Millipore (Darmstadt, Germany) and Sigma-Aldrich (Germany).

### Preparing of the essential oil

SKJ was obtained from a cultivated source (Khorraman Farm, Khorramabad, Iran) during the plant's flowering period, identified by the Department of Botany of the Research Institute of Forests and Rangelands (TARI; Tehran, Iran), and assigned a specimen voucher number (No. 58416) at the Herbarium of TARI. The obtained materials were crushed after drying in the air and then boiled in distilled water for 5 h using a Clevenger machine before being purified. The evaporated essence mixture and the water were separated based on the difference in mass between the water and the essence. A sodium sulfate solution was used to absorb the suspended water particles after the yellow oil (essence) was obtained. Carvacrol (91.33%), p-Cymene (2.29%), and l-Linalool (0.68%) made up the majority of the essence, according to previous gas chromatography-mass spectroscopy (GC-Mass) analysis^16,21^ (Supplemental Fig. 2). All procedures of collecting, boiling, preparing of essential oil and determining its component were done according to the Khorraman Company standard guideline. The essential oil was diluted in 1% Tween^®^ 20 and administered 30 min after TBI induction, similar to previous studies^13^.

### Diffuse traumatic brain injury (TBI) induction

All rats were intubated before the TBI induction. Based on the Marmarou approach^36–38^, the adopted TBI method was a moderate diffuse brain injury. Briefly, all the animals were anesthetized with ketamine and xylazine in preparation for TBI. Then, a 300 g weight was dropped from a height of 2 m onto the anesthetized rat's head, while a metal disc (10 mm in diameter, 3 mm thick) was affixed to the animal’s skull. After trauma induction, all rats were immediately connected to the breathing pump (Animal Respiratory Compact, TSE systems, Germany). The intra-tracheal tube was removed once the spontaneous breathing had returned, and rats were housed in individual cages. The site of brain damage was clearly visible on H&E-stained slides (Supplemental Fig. 3).

### Brain water content (BWC) determination

Each rat BWC was measured 24 h after the TBI induction to quantify cerebral edema. As previously mentioned, anesthetized rats were sacrificed, and their brains were rapidly removed and subjected to wet-dry weight comparisons to measure water content percent (%). Briefly, the brain was weighed after placement in pre-weighed glass vials (wet weight). Then, the vials were placed in a 100 °C incubator (Memmert, Germany) for 24 h before being weighed again (dry weight). The percentage of water in each brain specimen was then calculated using the following formula^39^:

$${\text{Brain water content }}\left( \% \right) \, = \, \left[ {\left( {{\text{wet weight}} - {\text{dry weight}}} \right)/{\text{wet weight}}} \right] \, \times { 1}00$$

### Neurological outcomes assessment

The veterinary coma scale (VCS) (3–15), the sum of the respiratory response score (1–3), the motor response score (1–8), and the visual response score (1–4) were used to assess neurological outcomes. The definitions of the stated scoring systems are presented in Table 2. A higher score indicates better neurological outcomes. The VCS was measured before TBI induction and 4 and 24 h afterward^40^.

**Table 2:** Veterinary coma scale.

Veterinary coma scale variable	Score
Motor function	
Normal movement	8
Mildly drowsy with spontaneous purposeful movements	7
Lethargic, unable to stand, but maintains sternal recumbency	6
Lethargic, withdraws to pinch, and lifts head with attention to visual stimuli; no sternal recumbency	5
Withdraws or pedals to pinch	4
Spontaneous pedaling	3
Extensor posturing (spontaneous or to stimuli)	2
Flaccid to stimuli	1
Eye function	
Open	4
Open on stimulation	3
Normal eyelid reflexes	2
No eyelid response to stimuli	1
Respiration	
Normal	3
Ataxic	2
Apneic	1

### Western blot analysis of apoptotic and inflammatory markers

The brain was removed from the skull and divided into two hemispheres. The right hemispheres of the brain were homogenized in 700 μl ice-cold RIPA lysis buffer (sigma; R0278), 1 mM protease inhibitors (sigma; P2714-IBTL), and 1 mM sodium orthovanadate using a tissue homogenizer (Hielscher UP200-Germany). The homogenate was centrifuged at 17,000×*g* for 15 min at 4 °C^13,41,42^. The supernatant was collected, and its protein concentrations were determined using the Bradford method (Bio-Rad Laboratories, München, Germany)^41^. On a 10% SDS-PAGE gel, equal amounts of protein (40 g) were separated electrophoretically and transferred to nitrocellulose membranes (Hybond ECL, GE Healthcare Bio-Sciences Corp., NJ, USA). The membranes were blocked for 2 h with 5% non-fat milk, then incubated overnight at 4 °C with the following antibodies; Bax (1:200, B-9: sc-7480), Bcl-2 (1:200, N-19: sc-492), NF-κB p65 (1:200, F-6: sc-8008), and caspase-3 (1:200, E-8: sc-7272). The membranes were incubated for 2 h at room temperature with the secondary antibodies (1:5000, mouse anti-rabbit IgG-HRP: sc-2357) and (1:5000, m- IgGκBP-HRP: sc-516102) followed by three times washing with tris-buffered saline with Tween^®^ (TBST; 15 min each). All antibodies were diluted in a blocking buffer containing 5% non-fat dried milk and TBST. Control of loading was performed using β-actin immunoblotting (antibody from Cell Signaling Technology Inc., Beverly, MA, USA; 1:200). Antibody-antigen complex was visualized via the Enhanced Chemiluminescence (ECL) method using the Gel Documentation System (BIO-RAD). The density of the bands was calculated using Lab Works software^43,44^.

### Enzyme-Linked immunosorbent assay (ELISA) of inflammatory cytokines

The tissue levels of TNF-α, IL-1β, and IL-6 were determined using commercially available ELISA kits (Eastibiopharm, USA). Briefly, the left cerebral hemispheres of rats were extracted and homogenized in phosphate buffer (pH 7.4, 0.1 M) using a homogenizer (Hielscher UP200-Germany) 24 h after TBI induction. The samples were then centrifuged at 3500 rpm for 15 min at 4 °C. The supernatant was collected and kept at − 80 °C until the analysis of TNF-α, IL-1β, and IL-6 levels as described by the kits manufacturer’s instructions^45^.

### Immunohistochemistry (IHC)

To determine neuronal activation, neuron-specific enolase (NSE) levels were assessed^46^ at 24 h after injury in paraffin-embedded sections (4 μm) using a Leica DM500-Germany microscope and ICC50 HD digital camera. Positive IHC-stained cells were counted in five different high-power fields (HPF; 40X). Neuron-specific enolase (NSE) IHC (NSE-IHC) protocols in this lab have been previously published^13^.

### Statistical analysis

To test the hypothesis that acute CAR treatment after diffuse TBI can reduce inflammatory and apoptotic properties at 24 h, associated with CARs ability to improve VCS scores similar to our previous publication testing SKEO^13^, statistical analyses were performed using SPSS, version 24.0 (SPSS, Inc., Chicago, IL, USA). Independent data are in structured data sets that are normally distributed (Shapiro–Wilk test) with similar variances (Brown-Forsythe test) meeting assumptions for the use of analysis of variance (ANOVA)^47,48^. One-way analysis of variance (ANOVA) was used to examine BWC and cytokines data, followed by a Tukey’s honest significance test (HSD) for post-hoc analysis. Repeated measure ANOVA was done on the logarithmic transformation of VCS scores to compare VCS scores between groups at different times by Bonferroni post-hoc test, similar to previous publications from our group^49^. Protein density and IHC data were compared between studied groups using one-way ANOVA followed by Tukey’s post-hoc test. Data are presented as the mean ± the standard error from the mean (SEM). Differences were considered statistically significant at the *P* < 0.05 level.

## Results

### SKEO and CAR treatments improved veterinary coma scale (VCS) scores after TBI

VCS changes in trial groups at different times after TBI are shown in Table 3. There were no significant differences in VCS between groups before TBI. A significant decrease was shown in the VCS scores in the TBI group at 4 h (9.33 ± 0.33) and 24 h (11.33 ± 0.33) post-TBI in comparison with the sham group (15 ± 0.0) (*P* < 0.001 and *P* < 01, respectively). The VCS scores in the SKEO (200 mg/kg) group at 4 h (12.00 ± 0.58) and 24 h (13.17 ± 0.31) post-TBI increased as compared to the Veh group (*P* < 0.01 and ns, respectively). Also, VCS scores in the CAR (200 mg/kg) group significantly increased at 4 h (12.33 ± 0.33) and 24 h (14.5 ± 0.22) post-TBI compared to the Veh group (*P* < 0.05 and *P* < 0.05, respectively). There was no significant difference in VCS between the TBI and TBI + Veh groups at any time after TBI.

**Table 3:** Veterinary coma scale (VCS) scores.

Animal groups	VCS (-1)	VCS (0)	VCS (4)	VCS (24)
Sham	15 ± 0	15 ± 0	15 ± 0	15 ± 0
TBI	15 ± 0	15 ± 0	9.33 ± 0.33^###^	11.33 ± 0.33^##^
TBI + Veh	15 ± 0	15 ± 0	9.17 ± 0.31^###^	11.00 ± 0.37^##^
CAR (100 mg/kg)	15 ± 0	15 ± 0	9.33 ± 0.17^###^	11.67 ± 0.49^#^
CAR (200 mg/kg)	15 ± 0	15 ± 0	12.33 ± 0.33^#^*	14.50 ± 0.22*
SKEO (200 mg/kg)	15 ± 0	15 ± 0	12.00 ± 0.58**	13.17 ± 0.31^#^

Data are expressed as mean ± SEM.

TBI: traumatic brain injury; SKEO: Satureja Khuzistanica essential oil; CAR: Carvacrol.

^#^P < 0.05, ^##^P < 0.01 & ^###^P < 0.001 represents significant difference with sham; *P < 0.05,*P < 0.01,*P < 0.001 represents significant difference with TBI + Veh.

### SKEO and CAR treatments reduced brain edema after TBI

**Figure 1:**
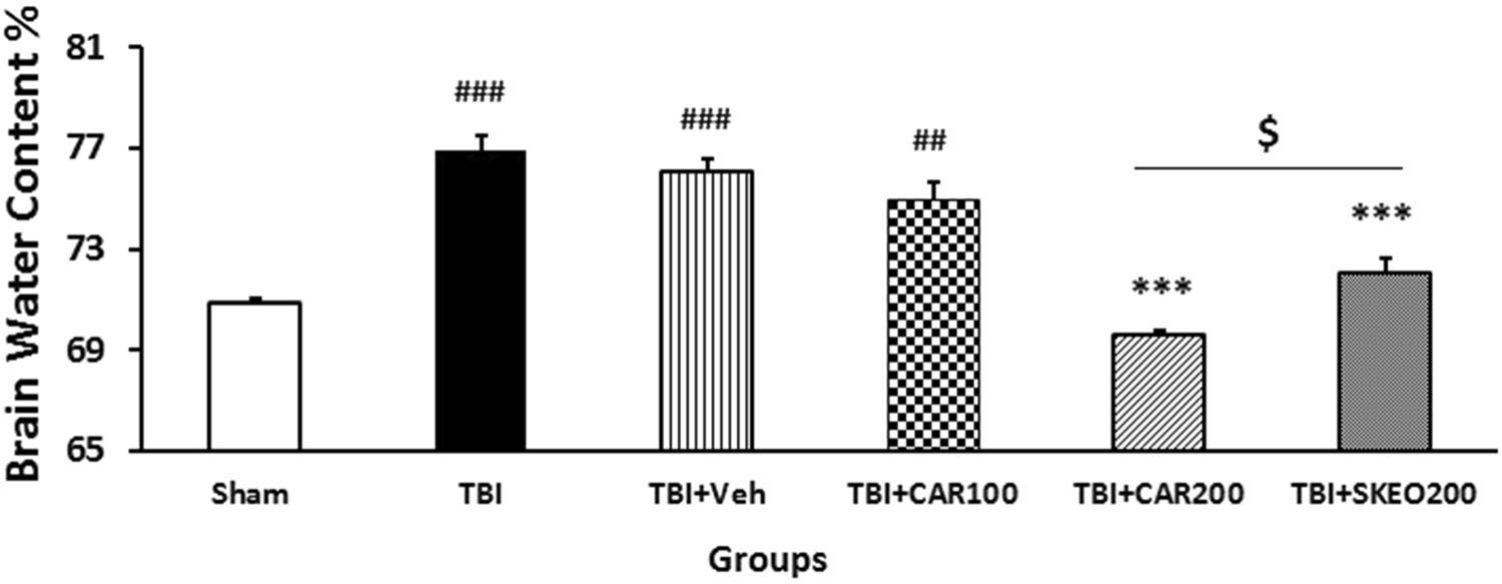
Effects of SKEO and CAR on the percent of brain water content 24 h after TBI induction in the different experimental groups (n = 6 rats per group). Data are expressed as mean ± SEM. ^##^*P* < 0.01 and ^###^*P* < 0.001 represents significant difference with sham; ***P < 0.001 represents significant difference with TBI + Veh; and ^$^*P* < 0.05 represents significant difference with TBI + SKEO. TBI: Traumatic brain injury; SKEO: *Satureja khuzistanica* essential oil; CAR: Carvacrol.

Changes in BWC 24 h post-TBI have been shown in Fig. 1. The BWC increased from 70.83 ± 0.23 to 76.88 ± 0.58 and 76.02 ± 0.51 in the TBI and TBI + Veh groups compared to the sham group (*P* < 0.001). The BWC in the TBI + SKEO200 and TBI + CAR200 groups was significantly lower than in the TBI + Veh group (*P* < 0.001), whereas there was no significant difference between the TBI and TBI + Veh groups. The administration of CAR100 had no significant effects on BWC. The BWC was significantly decreased in the TBI + CAR200 group (69.55 ± 0.78) compared to the TBI + SKEO200 group (72.02 ± 0.59) (*P* < 0.05). Based on the dose–response investigation, we discovered that a 100 mg/kg CAR dosage was ineffective, but a 200 mg/kg dose significantly decreased BWC (Fig. 1) and improved VCS at 4 and 24 h after TBI (Table 3).

### Effects of SKEO and CAR on the apoptotic-related proteins after TBI induction

**Figure 2:**
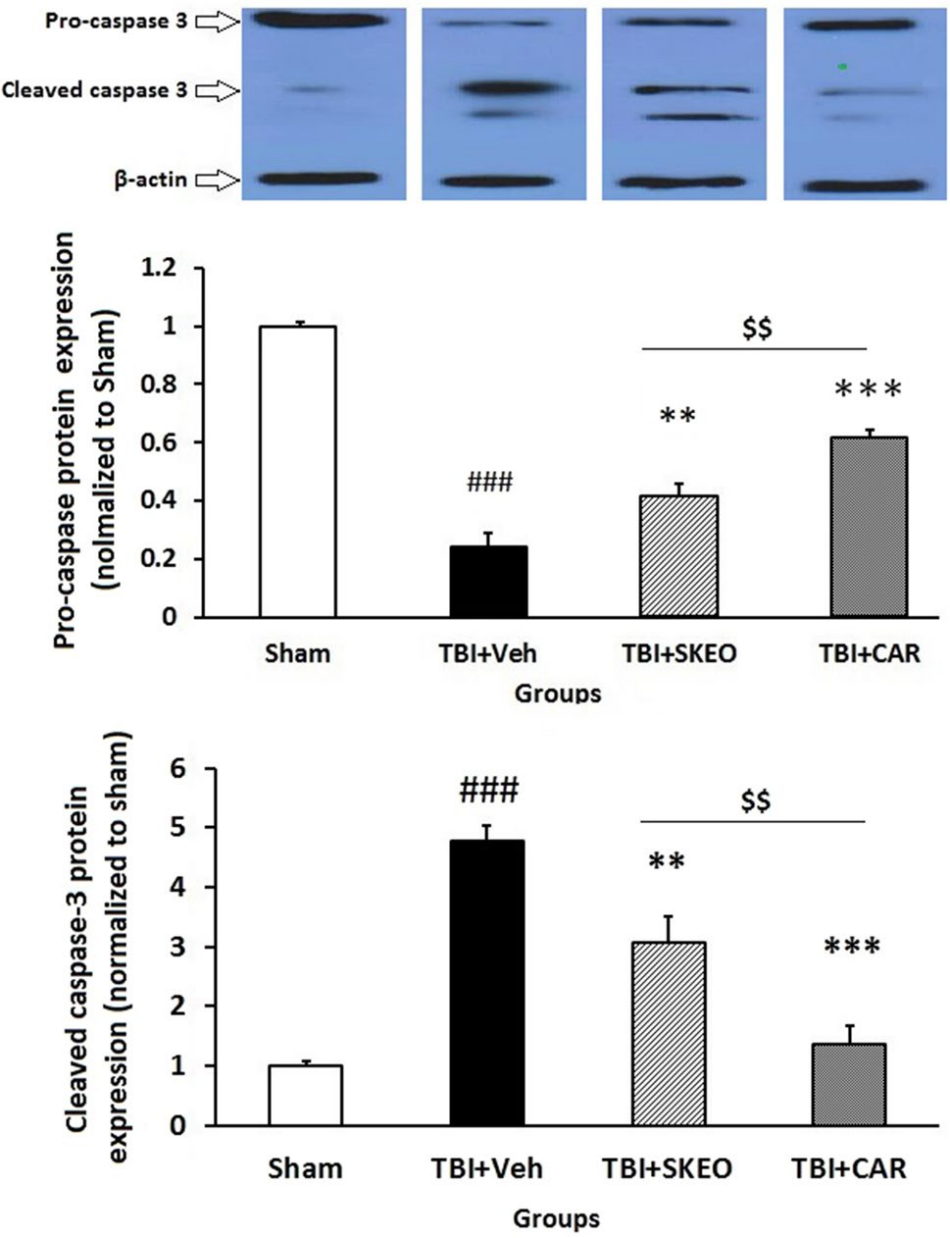
Effects of SKEO and CAR on the cleaved caspase-3 protein expression were assessed by western blotting 24 h after TBI induction in the different experimental groups (n = 6 rats per group). Cropped representative bands from the western blot test from the same run are shown above. Data are expressed as mean ± SEM. ^###^*P* < 0.001 represents significant difference with sham; **P < 0.01 and ***P < 0.001 represent significant differences with TBI + Veh; ^$$^*P* < 0.01 represents significant difference between TBI + CAR and TBI + SKEO. TBI: Traumatic brain injury; SKEO: *Satureja khuzistanica* essential oil; CAR: Carvacrol.

The proenzyme (32 kDa) and the active fragment (17 kDa) of caspase-3 were evaluated using the western blot technique. The proactive form of caspase-3 was reduced in the brain 24 h after injury, suggesting its possible conversion to the active form. Cleaved Caspase-3 protein expression was significantly elevated in the vehicle-treated animals (TBI + Veh) (4.8 ± 0.25, F = 35.42) compared to the sham group (*P* < 0.001) and was significantly diminished after CAR200 (1.53 ± 0.3) (*P* < 0.001) and moderately suppressed by SKEO200 (3.08 ± 0.4) (*P* < 0.01) (Fig. 2).

**Figure 3:**
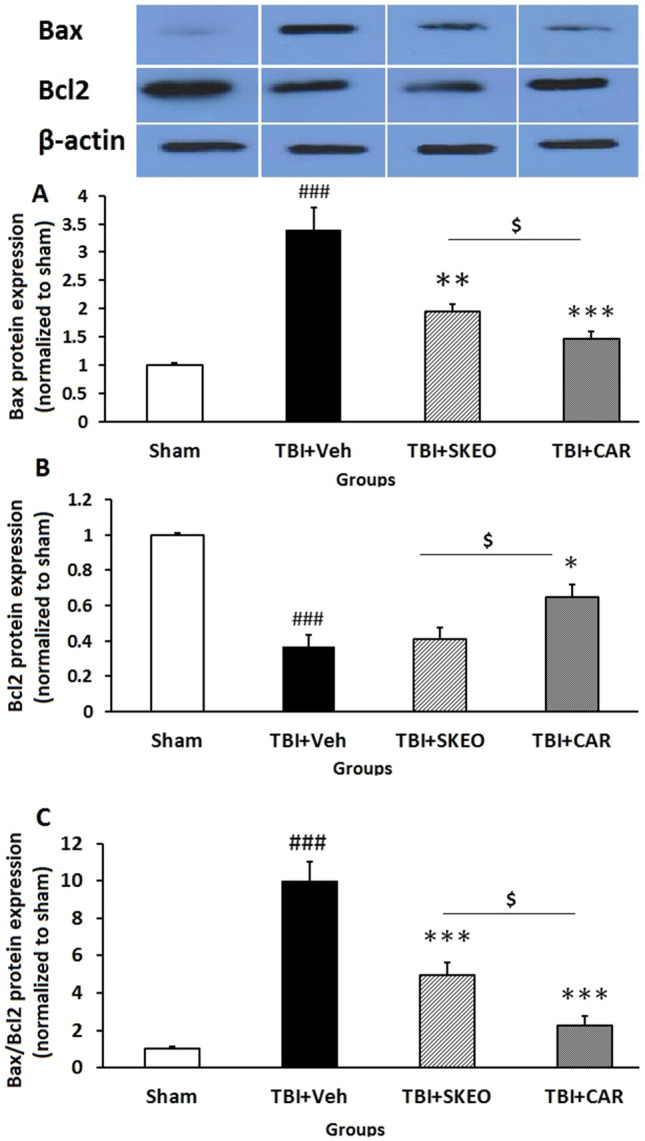
Effects of SKEO and CAR on the (**A**) Bax, (**B**) Bcl-2 protein levels, and (**C**) Bax:Bcl-2 ratio 24 h after TBI induction in the different experimental groups (n = 6 rats per group). Cropped representative bands from the western blot test from the same run are shown above. Data are expressed as mean ± SEM band density ratio for each group. β-actin was used as an internal control. ^###^*P* < 0.001 represents significant difference with sham; *P < 0.05, **P < 0.01, and ***P < 0.001 represent significant differences with TBI + Veh; ^$^*P* < 0.05 represent significant differences between TBI + CAR and TBI + SKEO. TBI: Traumatic brain injury; SKEO: *Satureja khuzistanica* essential oil; CAR: Carvacrol.

Expression of apoptotic-related proteins (Bax and Bcl-2) was used to investigate the protective effects of SKEO and CAR against TBI-induced neuronal death. The proapoptotic factor Bax (26 kDa) expression was low in the brains of sham rats, but the level of Bax expression in TBI + Veh was increased at 24 h post-TBI (3.11 ± 0.75, F = 18.74, P < 0.001) compared to sham group as shown in Fig. 3A. After SKEO and CAR treatment, Bax protein expression levels were significantly reduced (1.94 ± 0.13, P < 0.01) (1.44 ± 0.12, P < 0.001); respectively. Our findings indicated that SKEO and CAR administration was capable of modulating Bax expression following TBI induction, where CAR was more effective at lowering proapoptotic Bax protein compared to SKEO (P < 0.05) in this TBI model (Fig. 3A).

Moreover, the Western blot analysis showed that he expression of Bcl-2 was dramatically decreased in the TBI + Veh group (0.32 ± 0.06, F = 30.95, P < 0.001) 24 h after injury compared to the sham group, but this effect was significantly restored in the TBI-induced rats treated with CAR (0.68 ± 0.07, P < 0.05) but not by SKEO (0.41 ± 0.06) (Fig. 3B).

Furthermore, the Bax:Bcl-2 ratio alterations were compared between the studied groups. Injured animals in the TBI + Veh group (9.99 ± 1.01) had a significantly higher Bax:Bcl-2 protein ratio than those in the sham group (*P* < 0.001). The elevated Bax:Bcl-2 ratio was significantly reduced in injured rats treated with 200 mg/kg SKEO (4.96 ± 0.67) and CAR (2.26 ± 0.48) compared to the TBI + Veh group (P < 0.01 and *P* < 0.001, respectively) (Fig. 3C). There was a significant difference between CAR and SKEO treatment groups (P < 0.05).

### SKEO and CAR reduced the inflammatory cytokines after TBI induction

**Figure 4:**
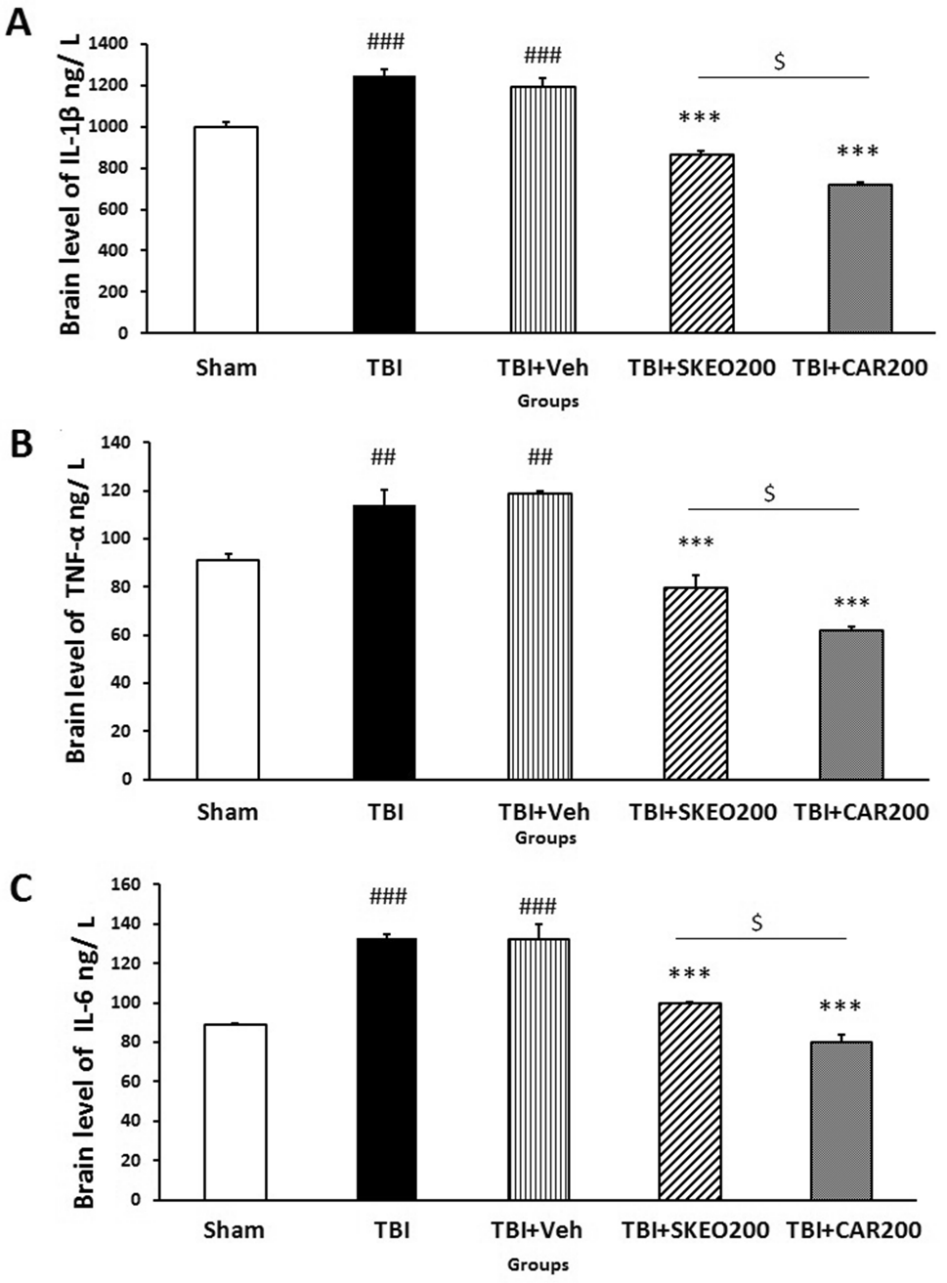
Effects of SKEO and CAR on the (**A**) IL-1β, (**B**) TNF-α, and (**C**) IL-6 levels 24 h after TBI induction in the different experimental groups (n = 6 rats per group). Data are expressed as mean ± SEM. ^##^*P* < 0.01 & ^###^*P* < 0.001 represent significant differences with sham; ***P < 0.001 represents significant difference with TBI + Veh; and ^$^*P* < 0.05 represents significant difference between TBI + CAR and TBI + SKEO. TBI: Traumatic brain injury; SKEO: *Satureja khuzistanica* essential oil; CAR: Carvacrol.

The tissue levels of IL-1β, TNF-a, and IL-6 in rat brains were significantly increased 24 h after TBI induction compared to the sham group. No significant differences were observed between the TBI and TBI + Veh groups. Figure 4A indicates a significant decrease in IL-1β levels in the TBI + SKEO (862.33 ± 21.88) and TBI + CAR (715.66 ± 12.99) groups compared to the TBI + Veh (1186.6 ± 46.75) group (*P* < 0.001). There was also a significant difference between the TBI + SKEO and TBI + CAR groups (*P* < 0.05).

TNF-α levels were also decreased significantly in the TBI + SKEO (79.61 ± 5.2) and TBI + CAR (62.08 ± 1.5) groups (*P* < 0.001) compared to the TBI + Veh group (118.89 ± 1.23) (Fig. 4B). A significant difference in TNF-α levels was found between the TBI + SKEO and TBI + CAR groups (*P* < 0.05).

Figure 4C also shows that SKEO (99.68 ± 0.42) and CAR (80.13 ± 3.83) treatment after TBI induction significantly decreased the level of IL-6 compared to the TBI + Veh group (132.25 ± 7.81) (*P* < 0.001). The results also showed that CAR significantly reduced IL-6 levels compared to the SKEO (*P* < 0.05).

**Figure 5:**
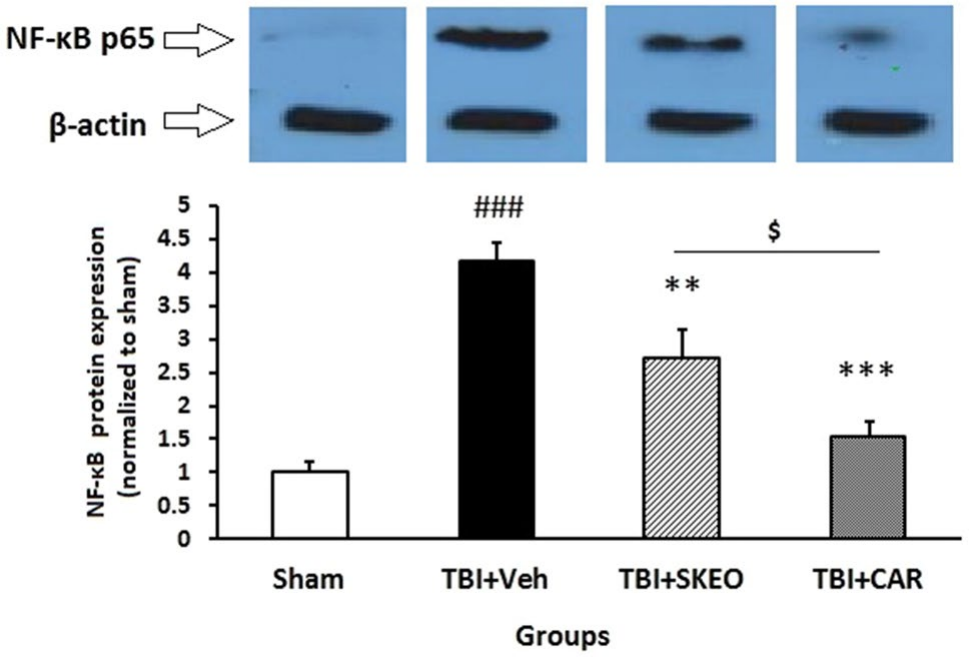
Effects of SKEO and CAR on the inflammatory protein NF-κB p65 expression 24 h after TBI induction in the different experimental groups (n = 6 rats per group). Cropped representative bands from the western blot test from the same run are shown above. Data are expressed as mean ± SEM band density ratio for each group. β-actin was used as an internal control. ^###^*P* < 0.001 represents a significant difference with sham; **P < 0.05 and ***P < 0.01 represent significant differences with TBI + Veh. TBI: Traumatic brain injury; SKEO: *Satureja khuzistanica* essential oil; CAR: Carvacrol.

Western blot assays were used to determine whether the neuroprotective effect of CAR and SKEO was due to the inhibition of NF-κB signaling. TBI substantially enhanced NF-κB p65 expression (4.18 ± 0.26) compared to the sham group (P < 0.05). In contrast, SKEO (2.72 ± 0.41) and CAR (1.53 ± 0.22) administration significantly reduced the postinjury NF-*κ*Bp65 protein expression in the brain when compared to the TBI + Veh group (*P* < 0.01 & *P* < 0.001, respectively) (Fig. 5).

### SKEO and CAR inhibited neuron injury

**Figure 6:**
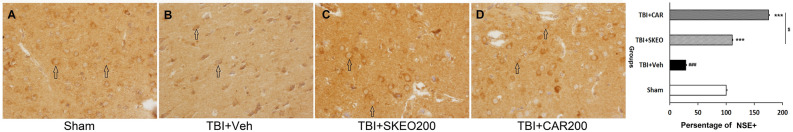
(**A**–**D**) IHC staining for NSE in the rat brain (×40). (**A**) Sham; arrow indicates the normal neurons. (**B**) 24 h after TBI; arrow indicates the degenerated neurons. (**C**,**D**) TBI + SKEO200& TBI + CAR200; arrow indicates the viable neurons. The scale bar illustrates the means ± SEM percentages of neuron-specific enolase (NSE from different groups. ^###^p < 0.001 compared to the sham group, ***p < 0.01 compared to the TBI group, and ^$^*P* < 0.05 represents significant difference between TBI + CAR and TBI + SKEO. TBI: Traumatic brain injury; SKEO: *Satureja khuzistanica* essential oil; CAR: Carvacrol.

We performed IHC staining for NSE to assess for neuronal loss/injury. NSE-IHC staining showed that the neuronal nucleus and the intact, brown-stained neural cytoplasm and membrane were present in neurons in the sham group (100 ± 1.55%). In contrast, in the TBI + Veh group, a few non-injured neurons were seen, accompanied by a large number of malformed or squeezed neurons in comparison with the sham group (28.42 ± 1.33%) (P < 0.001). However, treatment with SKEO and CAR, respectively, resulted in the survival of (110.71 ± 1.2%) and (175.02 ± 1.62%) of neurons, positively stained, in comparison with the TBI + Veh group (P < 0.001). The results also showed that CAR potently and significantly reduced neural damage compared to the SKEO (*P* < 0.05) (Fig. 6).

## Discussion

In the present study, we established a successful TBI weight-drop model and confirmed it by evaluating the rate of brain edema, neurological scores, and histological findings (Figs. 1, 6, Table 3, and Supplemental Fig. 1). Furthermore, the current study findings revealed that SKEO and CAR could reduce edema and have beneficial effects on regulating apoptotic and inflammatory signaling pathways following TBI model induction. Administration of SKEO and CAR reduced pro-apoptotic proteins such as Bax and caspase-3 and improved anti-apoptotic proteins such as Bcl-2 (Figs. 2 and 3). Moreover, after brain injury induction, the inflammatory signaling molecules, such as the NF-κB, IL-1β, TNF-α, and IL-6, were significantly reduced following CAR and SKEO treatments (Figs. 4 and 5). It should be noted that in most of the studied variables, the effects of CAR were more potent and significant than the SKEO.

Our previous findings showed that SKEO (200 mg/kg), which contains more than 94% CAR, inhibits astrocytes, neurons, and blood origin-infiltrated cells, all of which are cytokines-producing sources, lowering pro-inflammatory cytokines such as IL-1β, TNF-α, and IL-6, and resulting in considerably reduced brain injuries^13^.

The inflammatory response, which plays a crucial role in developing secondary damage such as cell death, is related to the generation of pro-inflammatory cytokines. Acute reduction in the inflammatory response has been implicated in reducing the morbidity and mortality associated with trauma^50^. We found significant neuronal cell death after TBI in this work, as demonstrated by alterations in VCS scores, elevated cleaved caspase-3 along with neuron damage (NSE-IHC) (Table 3, Figs. 2 and 6), consistent with earlier findings^10,51,52^, while SKEO and CAR therapy mitigated these changes.

The overall pathophysiology of TBI in humans and animals is associated with apoptosis or programmed cell death of neurons and glia^53^ and is regulated by several interconnected mechanisms, including extrinsic and intrinsic signaling pathways. In the weight drop TBI model, stimulation of the cell-extrinsic pathway triggers the binding of ligands, such as TNF-α and FasL, to their receptors on the cell surface, whereas higher permeabilization of Bax/Bak channels on the outer mitochondria membrane are the central events in the intrinsic pathway, which leads to the cytochrome c release, a protein that plays a crucial role in cell death and is typically inhibited by the Bcl-2 protein. Then, cytochrome c forms the apoptosome complex in the cytosol. These two mechanisms converge at the level of effector caspases, such as caspase-3 and caspase-7, resulting in the cleavage of cellular proteins and apoptosis^51,54–56^. It has also been demonstrated that decreasing caspase-3 activation and apoptotic cell death promotes functional recovery in animals following TBI^57,58^.

Anti-apoptotic genes like Bcl-2^59^ and pro-apoptotic genes like Bax^60^ belong to the Bcl-2 multigene superfamily. Following localized and global ischemia and TBI, damaged neurons showed decreased Bcl-2 and elevated Bax immunoreactivity^61–63^. The ratio of anti-apoptotic and pro-apoptotic Bcl-2 superfamily proteins appears to be essential for cell survival following CNS damage and in other cells^20,64–68^. After TBI in rat brains, changes in the cellular Bax:Bcl-2 ratio may mediate cell death via modulating the activity of the cell death-inducing caspase family of proteases^69,70^.

In the present study, TBI induces an increase in the Bax:Bcl-2 ratio in the TBI + Veh group, whereas SKEO and CAR treatments cause a more substantial decline in the Bax:Bcl-2 ratio in rat brains (Fig. 3). As a result, the decrease in the Bax:Bcl-2 protein ratio in the brains of rats treated with SKEO and CAR may be critical for reducing cleaved caspase-3 synthesis and, consequently, neurological scores improvement. Consistent with these results, Peter et al.^32^ reported neurological function improvement after CAR administration in a mouse model of weight drop closed head injury. Another research study showed that administration of *Satureja khuzistanica* extract reduced motor deficits in diabetic rats, which has been associated with a reduction in cleaved caspase-3 protein and Bax:Bcl-2 ratio in rat dorsal root ganglion^21^.

There is growing evidence that pro-apoptotic family members promote cytokine release into the cytoplasm, which leads to caspase activation^10–12^. CAR-rich SKEO has been demonstrated to reduce anti-inflammatory and neuroprotective properties, but the full mechanistic range of CAR and SKEO remains unknown^31^. Therefore, a rat model was used to investigate whether SKEO and CAR may help prevent neuroinflammation-induced apoptosis following TBI. Findings of our previous study^71^ and current results showed that TBI induced inflammation in the rat brains by overexpressing IL-1β, TNF-α, and IL-6, while SKEO200 and CAR200 treatment alleviated these inflammatory markers (Fig. 4). According to Li et al. findings, CAR decreased the levels of TNF-α and IL-1β, as well as the production of inducible nitric oxide synthase (iNOS) and cyclooxygenase-2 (COX-2) in ischemic cortical tissues. They also found that CAR inhibited tissue invasion by cellular markers of inflammatory enzymes^72^.

Microglia generate members of the pro-inflammatory cytokine family, which raise BBB permeability^73^. Brain edema and neuronal degeneration were linked to increased pro-inflammatory cytokines^74^. The findings of the present study, in agreement with our previous study^11^, demonstrated an increase in cerebral edema accompanied by up-regulation levels of pro-inflammatory cytokines following TBI in rat brains. The ability of CAR to minimize edema has been shown in previous research, which supports CAR to diminish edema and inflammatory responses by suppressing TNF-α^75^. Zhong et al. demonstrated the preventive effects of CAR on an intracerebral hemorrhage by reducing cerebral edema^33^. In the current study, CAR200 dramatically reduced cerebral edema and levels of pro-inflammatory cytokines, IL-1β, TNF-α, and IL-6. In our previous study, the Evans blue dye extravasation was used to quantify BBB permeability and revealed that SKEO200 reduced the water accumulation in the brain parenchyma due to improved vascular consistency^13^. Accordingly, we predict that the reduction in cerebral edema in the SKEO-treated group in this study was probably through a similar vascular mechanism (Fig. 1). Besides this, our recent finding showed that CAR potentially protects BBB via zonula occludens-1(ZO-1)/occludin, tight junction proteins, in the rat^76^. Consistent with our results, Park and colleagues revealed the efficacy of CAR in reducing vascular permeability following spinal cord injury by raising the tight junction and adhesion proteins levels^77^. Increased BBB permeability allows blood cells such as neutrophils, lymphocytes, monocytes, and macrophages to enter the central nervous system^78^, followed by releasing mediators such as prostaglandins, free radicals, and inflammatory cytokines, which trigger the production of chemokines and adhesion molecules and activate immune cells and glia^79^. As a result, it appears that a reduction in vascular permeability is one of the reasons for the reduction of edema and inflammatory cytokines by CAR in the current study.

TNF-α not only promotes the expression of other interleukins, like IL-6, which cause nerve impairment, BBB damage, and acute neurological problems in injury sites^79,80^, but it can also trigger programmed cell death in neurons^81,82^. Cytokines also have a role in apoptosis and inflammation by degrading IkappaB kinase (IκB) and activating the NF-κB signaling pathway, which is critical in initiating apoptosis^83^. NF-κB is a proinflammatory regulatory cytokine that can enhance the effects of other inflammatory signals^84^. Due to binding to the IκB inhibitor, the NF-κB transcription factor complex is generally retained in the cytoplasm in its inactive form. Upon activation, IκB is ubiquitinated and degraded, allowing the NF-B complex to be phosphorylated and transported into the nucleus, where it can bind to promoter sites to regulate the transcription of target genes^85^. Nearly the whole arsenal of immunological defenders is triggered by NF-κB, including chemokines, cytokines, adhesion molecules, inflammatory mediators, and apoptosis inhibitors, giving NF-κB a crucial role in global immunity^86^ that is conserved across species^87^. NF-κB is also implicated in the downregulation of pro-survival brain-derived neurotrophic factor (BDNF) at 24 h following exposure to high extracellular glutamate levels^88^, a common occurrence after mechanical depolarization during TBI. In an experimental model of closed-head injury, repression of the NF-κB inhibitor system enhanced neuronal cell death, worsened neurological outcomes, and accelerated post-traumatic mortality rate. It has been observed that NF-κB activation continues for a long time in glial cells and neurons following TBI, and it might regulate pro-apoptotic factors such as Bax protein^89^. In this regard, Li et al. reported the inhibitory effect of CAR on the NF-κB p65 (active form) that was generated after ischemia–reperfusion in rat brains^72^. In the present study, SKEO and CAR treatments efficiently reduced NF-κBp65 protein expression, indicating that the plant-origin essential oil, SKJ, and commercial CAR may attenuate post-traumatic inflammatory responses by modifying NF-κB signaling (Fig. 5) by decreasing Bax protein and ultimately caspase-3 protein activation causing improved VCS. Because CAR is the suspected principal active component in SKEO, it is plausible to assume that CAR is responsible for SKEO protective effects on post-traumatic outcomes and that the reduction in NF-κBp65 protein in the TBI + SKEO group was related to its CAR content performance. While NF-κB signaling is predominantly associated with pathological processes, there is growing evidence that it can mediate neuroprotective pathways through the upregulation of hemoxygenase 1 (HO-1), early growth response protein 1 (Egr-1), and heat shock protein β-1 of 27 KB (Hsp27) after excitotoxicity and stroke^88,90^, which requires further consideration for the impact on NF-κB signaling associated with chronic inflammation after experimental TBI^91–94^.

In the present study, although SKEO contains a significant amount of carvacrol (91.33%), it showed weaker effects than pure CAR. In general, essential oils have multifunctional activities due to the presence of various components. Numerous comparative studies have examined the effects of essential oils with the most constituent components. They indicated that the active substances are not necessarily more potent than the essential oils. For example, Magierowicza et al. investigated the biopesticide activity of *Satureja hortensis* essential oil containing mainly CAR (73.24%) and purified CAR. They found that the essential oil was more effective than its active ingredients alone^95^. Another comparative study showed that CAR-rich SKEO (87.16%) has the same antimicrobial properties as commercial CAR^96^.

Extensive research has shown that the activity of essential oils is influenced by various factors such as seasonal variation and harvesting month. These factors are so important that they can lead to changes in potency and poor performance of the essential oil or existing compounds. For example, it is said that the famous essential oil called Ocimum gratissimum, popularly known as basil essential oil, has variable activities on the mouse central nervous system depending on the harvest season and essence extraction^97^. Other environmental factors can also affect the activity of essential oils, such as day/night temperature, rainfall and intensity of sunlight^98^. Fortunately, many of the above factors are compensable or adjustable^99^. Therefore, the difference between the TBI + SKEO and TBI + CAR groups in our study may be due to the aforementioned interfering factors.

According to the results found in the present study, it can be concluded that SKEO and CAR can reduce acute edema and inhibit the production of pro-inflammatory cytokines regulator NF-κB and apoptotic-related proteins caspase 3, resulting in the preservation of neuronal death and acute neurological functions in this model of TBI. The long-term outcomes require further investigation to confirm if a single treatment of SKEO or CAR can prevent persisting TBI-induced symptoms.

## Supplementary Information

Supplementary Legends.

Supplementary Figure 1.

Supplementary Figure 2.

Supplementary Figure 3.

Supplementary Figure 4.

## Data Availability

The datasets used and/or analysed during the current study are available from the corresponding author on reasonable request.

## References

[1] Hyder AA, Wunderlich CA, Puvanachandra P, Gururaj G, Kobusingye OC (2007). The impact of traumatic brain injuries: A global perspective. NeuroRehabilitation.

[2] Global status report on road safety 2018: summary. Geneva: World Health Organization; (WHO/NMH/NVI/18.20). License: CC BY-NC-SA 3.0 IGO). (2018).

[3] Dewan MC (2018). Estimating the global incidence of traumatic brain injury. J. Neurosurg..

[4] Williams J (2020). Cost-effectiveness analysis of tranexamic acid for the treatment of traumatic brain injury, based on the results of the CRASH-3 randomised trial: A decision modelling approach. BMJ Glob. Health.

[5] Thapa, K., Khan, H., Singh, T. G. & Kaur, A. Traumatic brain injury: Mechanistic insight on pathophysiology and potential therapeutic targets. *J. Mol. Neurosci.* **71**(9), 1725–1742 (2021). 10.1007/s12031-021-01841-7 33956297

[6] Jang C-H, Choi J-H, Byun M-S, Jue D-M (2006). Chloroquine inhibits production of TNF-α, IL-1β and IL-6 from lipopolysaccharide-stimulated human monocytes/macrophages by different modes. Rheumatology.

[7] Lenzlinger PM, Morganti-Kossmann M-C, Laurer HL, McIntosh TK (2001). The duality of the inflammatory response to traumatic brain injury. Mol. Neurobiol..

[8] Chio C-C (2017). Exercise attenuates neurological deficits by stimulating a critical HSP70/NF-κB/IL-6/synapsin I axis in traumatic brain injury rats. J. Neuroinflamm..

[9] Neurath MF, Pettersson S, ZumBüschenfelde K-HM, Strober W (1996). Local administration of antisense phosphorothiate olignucleotides to the p65 subunit of NF-κB abrogates established experimental colitis in mice. Nat. Med..

[10] Zhang MH (2018). Neuroprotective effects of dexmedetomidine on traumatic brain injury: Involvement of neuronal apoptosis and HSP70 expression. Mol. Med. Rep..

[11] Xue Z (2017). Calcium-sensing receptor antagonist NPS2390 attenuates neuronal apoptosis though intrinsic pathway following traumatic brain injury in rats. Biochem. Biophys. Res. Commun..

[12] Zhao W-Y (2017). Establishment of an ideal time window model in hypothermic-targeted temperature management after traumatic brain injury in rats. Brain Res..

[13] Abbasloo E (2016). The anti-inflammatory properties of *Satureja*
*khuzistanica* Jamzad essential oil attenuate the effects of traumatic brain injuries in rats. Sci. Rep..

[14] Hadian J, Hossein Mirjalili M, Reza Kanani M, Salehnia A, Ganjipoor P (2011). Phytochemical and morphological characterization of *Satureja*
*khuzistanica* Jamzad populations from Iran. Chem. Biodivers..

[15] Abdollahi M (2003). Antioxidant, antidiabetic, antihyperlipidemic, reproduction stimulatory properties and safety of essential oil of *Satureja*
*khuzestanica* in rat in vivo: A toxicopharmacological study. Med. Sci. Monit..

[16] Khosravinia H (2014). Hypolipidemic effects of carvacrol in relation with sex hormones in broiler chicken. Biotechnol. Anim. Husb..

[17] Khosravinia H (2015). Hypolipidemic effects of *Satureja*
*khuzistanica* essential oil in broiler chicken are realized through alteration in steroid hormones. Kafkas Univ. Vet. Fak. Derg..

[18] Saberi, A. *et al.**Satureja khuzestanica* extract elicits antinociceptive activity in several model of pain in rats. (2013).

[19] Amanlou M, Dadkhah F, Salehnia A, Farsam H, Dehpour AR (2005). An anti-inflammatory and anti-nociceptive effects of hydroalcoholic extract of *Satureja*
*khuzistanica* Jamzad extract. J. Pharm. Pharm. Sci..

[20] Al Seyedan A, Dezfoulian O, Alirezaei M (2020). *Satureja*
*khuzistanica* Jamzad essential oil prevents doxorubicin-induced apoptosis via extrinsic and intrinsic mitochondrial pathways. Res. Pharm. Sci..

[21] Kaeidi A (2013). *Satureja*
*khuzestanica* attenuates apoptosis in hyperglycemic PC12 cells and spinal cord of diabetic rats. J. Nat. Med..

[22] Ghazanfari G (2006). Biochemical and histopathological evidences for beneficial effects of *Satureja*
*khuzestanica* Jamzad essential oil on the mouse model of inflammatory bowel diseases. Toxicol. Mech. Methods.

[23] Pirbalouti, A. G. & Moalem, E. Variation in antibacterial activity of different ecotypes of *Satureja khuzestanica* Jamzad, as an Iranian endemic plant. (2013).

[24] Turgut K, Özyiğit Y, Tütüncü B, Sözmen EU (2017). Agronomic and chemical performance of selected *Origanum*
*dubium* Boiss. clones for industrial use. Turk. J. Agric. For..

[25] Trabace L (2011). Estrous cycle affects the neurochemical and neurobehavioral profile of carvacrol-treated female rats. Toxicol. Appl. Pharmacol..

[26] Melusova M, Slamenova D, Kozics K, Jantova S, Horvathova E (2014). Carvacrol and rosemary essential oil manifest cytotoxic, DNA-protective and pro-apoptotic effect having no effect on DNA repair. Neoplasma.

[27] Samarghandian, S., Farkhondeh, T., Samini, F. & Borji, A. Protective effects of carvacrol against oxidative stress induced by chronic stress in rat’s brain, liver, and kidney. *Biochem. Res. Int.* **2016**, 1–7 (2016). 10.1155/2016/2645237 PMC4745576 26904286

[28] Suntres ZE, Coccimiglio J, Alipour M (2015). The bioactivity and toxicological actions of carvacrol. Crit. Rev. Food Sci. Nutr..

[29] Hong DK (2018). Carvacrol attenuates hippocampal neuronal death after global cerebral ischemia via inhibition of transient receptor potential melastatin 7. Cells.

[30] Jiang ZS, Pu ZC, Hao ZH (2015). Carvacrol protects against spinal cord injury in rats via suppressing oxidative stress and the endothelial nitric oxide synthase pathway. Mol. Med. Rep..

[31] Zamanian, M. Y. *et al.* Carvacrol as a potential neuroprotective agent for neurological diseases: A systematic review article. *CNS Neurol. Disord.-Drug Targets (Formerly Curr. Drug Targets-CNS Neurol. Disord.)* (2021). 10.2174/1871527320666210506185042 33970850

[32] Peters M (2012). Carvacrol together with TRPC1 elimination improve functional recovery after traumatic brain injury in mice. J. Neurotrauma.

[33] Zhong Z (2013). Carvacrol alleviates cerebral edema by modulating AQP4 expression after intracerebral hemorrhage in mice. Neurosci. Lett..

[34] Abbasloo E (2016). The anti-inflammatory properties of *Satureja*
*khuzistanica* Jamzad essential oil attenuate the effects of traumatic brain injuries in rats. Sci. Rep..

[35] Haidar MA (2022). Mitoquinone helps combat the neurological, cognitive, and molecular consequences of open head traumatic brain injury at chronic time point. Biomedicines.

[36] O'Connor CA, Cernak I, Vink R (2005). Both estrogen and progesterone attenuate edema formation following diffuse traumatic brain injury in rats. Brain Res..

[37] Keshavarzi Z, Amiresmaili S, Shahrokhi N, Bibak B, Shakeri F (2021). Neuroprotective effects of auraptene following traumatic brain injury in male rats: The role of oxidative stress. Brain Res. Bull..

[38] Farahani F (2022). Possible involvement of female sex steroid hormones in intracellular signal transduction mediated by cytokines following traumatic brain injury. Brain Res. Bull..

[39] Meymandi MS (2018). Effects of pregabalin on brain edema, neurologic and histologic outcomes in experimental traumatic brain injury. Brain Res. Bull..

[40] King DR, Cohn SM, Proctor KG (2004). Changes in intracranial pressure, coagulation, and neurologic outcome after resuscitation from experimental traumatic brain injury with hetastarch. Surgery.

[41] Abdi E (2018). Expression of IGF-1, IL-27 and IL-35 receptors in adjuvant induced rheumatoid arthritis model. Iran J. Immunol..

[42] Rostamzadeh F (2017). Heterodimerization of apelin and opioid receptors and cardiac inotropic and lusitropic effects of apelin in 2K1C hypertension: Role of pERK1/2 and PKC. Life Sci..

[43] Abbasloo E, Najafipour H, Esmaeili-Mahani S (2016). Induction of antinociceptive tolerance to the chronic intrathecal administration of apelin-13 in rat. Neuropeptides.

[44] Abbasloo E, Najafipour H, Vakili A (2020). Chronic treatment with apelin, losartan and their combination reduces myocardial infarct size and improves cardiac mechanical function. Clin. Exp. Pharmacol. Physiol..

[45] Jiao L (2011). Edaravone alleviates delayed neuronal death and long-dated cognitive dysfunction of hippocampus after transient focal ischemia in Wistar rat brains. Neuroscience.

[46] Dash PK, Zhao J, Hergenroeder G, Moore AN (2010). Biomarkers for the diagnosis, prognosis, and evaluation of treatment efficacy for traumatic brain injury. Neurotherapeutics.

[47] Song H (2022). The AMPK-SIRT1-FoxO1-NF-kappaB signaling pathway participates in hesperetin-mediated neuroprotective effects against traumatic brain injury via the NLRP3 inflammasome. Immunopharmacol. Immunotoxicol..

[48] Pati, S. K. *et al.* In *Advanced Machine Learning Approaches in Cancer Prognosis: Challenges and Applications* (eds Nayak, J. *et al.*) 13–73 (Springer International Publishing, 2021).

[49] Krishna G (2020). Traumatic brain injury-induced sex-dependent changes in late-onset sensory hypersensitivity and glutamate neurotransmission. Front. Neurol..

[50] Wang D (2018). Dexmedetomidine attenuates traumatic brain injury: Action pathway and mechanisms. Neural Regen. Res..

[51] Li F, Wang X, Zhang Z, Zhang X, Gao P (2019). Dexmedetomidine attenuates neuroinflammatory–induced apoptosis after traumatic brain injury via Nrf2 signaling pathway. Ann. Clin. Transl. Neurol..

[52] Polcyn R (2017). Neuron specific enolase is a potential target for regulating neuronal cell survival and death: Implications in neurodegeneration and regeneration. Neuroimmunol. Neuroinflamm..

[53] Raghupathi R, Graham DI, McINTOSH TK (2000). Apoptosis after traumatic brain injury. J. Neurotrauma.

[54] Zhang X, Chen Y, Jenkins LW, Kochanek PM, Clark RS (2004). Bench-to-bedside review: Apoptosis/programmed cell death triggered by traumatic brain injury. Crit. Care.

[55] Cernak I, Chapman SM, Hamlin GP, Vink R (2002). Temporal characterisation of pro-and anti-apoptotic mechanisms following diffuse traumatic brain injury in rats. J. Clin. Neurosci..

[56] Xing P (2018). The protection effect and mechanism of hyperbaric oxygen therapy in rat brain with traumatic injury 1. Acta cirurgica brasileira.

[57] Lu D (2004). Atorvastatin reduces neurological deficit and increases synaptogenesis, angiogenesis, and neuronal survival in rats subjected to traumatic brain injury. J. Neurotrauma.

[58] Wu H (2008). Increase in phosphorylation of Akt and its downstream signaling targets and suppression of apoptosis by simvastatin after traumatic brain injury. J. Neurosurg..

[59] Boise LH (1993). bcl-x, a bcl-2-related gene that functions as a dominant regulator of apoptotic cell death. Cell.

[60] Oltval ZN, Milliman CL, Korsmeyer SJ (1993). Bcl-2 heterodimerizes in vivo with a conserved homolog, Bax, that accelerates programed cell death. Cell.

[61] Gillardon F (1996). Altered expression of Bcl-2, Bcl-X, Bax, and c-Fos colocalizes with DNA fragmentation and ischemic cell damage following middle cerebral artery occlusion in rats. Mol. Brain Res..

[62] Krajewski S (1995). Upregulation of bax protein levels in neurons following cerebral ischemia. J. Neurosci..

[63] Zhang W, Hong J, Zheng W, Liu A, Yang Y (2021). High glucose exacerbates neuroinflammation and apoptosis at the intermediate stage after post-traumatic brain injury. Aging.

[64] Korsmeyer SJ (1995). Regulators of cell death. Trends Genet..

[65] Moorjani N (2007). Upregulation of Bcl-2 proteins during the transition to pressure overload-induced heart failure. Int. J. Cardiol..

[66] Sakinah SS, Handayani ST, Hawariah LA (2007). Zerumbone induced apoptosis in liver cancer cells via modulation of Bax/Bcl-2 ratio. Cancer Cell Int..

[67] Cheng C-Y, Tang N-Y, Kao S-T, Hsieh C-L (2016). Ferulic acid administered at various time points protects against cerebral infarction by activating p38 MAPK/p90RSK/CREB/Bcl-2 anti-apoptotic signaling in the subacute phase of cerebral ischemia-reperfusion injury in rats. PLoS ONE.

[68] Chen W (2015). TRPM7 inhibitor carvacrol protects brain from neonatal hypoxic-ischemic injury. Mol. Brain.

[69] Thornberry NA, Lazebnik Y (1998). Caspases: Enemies within. Science.

[70] Raghupathi R (2003). Temporal alterations in cellular Bax: Bcl-2 ratio following traumatic brain injury in the rat. J. Neurotrauma.

[71] Khaksari M, Abbasloo E, Dehghan F, Soltani Z, Asadikaram G (2015). The brain cytokine levels are modulated by estrogen following traumatic brain injury: Which estrogen receptor serves as modulator?. Int. Immunopharmacol..

[72] Li Z, Hua C, Pan X, Fu X, Wu W (2016). Carvacrol exerts neuroprotective effects via suppression of the inflammatory response in middle cerebral artery occlusion rats. Inflammation.

[73] Allan SM, Rothwell NJ (2001). Cytokines and acute neurodegeneration. Nat. Rev. Neurosci..

[74] Ding K (2014). Melatonin reduced microglial activation and alleviated neuroinflammation induced neuron degeneration in experimental traumatic brain injury: Possible involvement of mTOR pathway. Neurochem. Int..

[75] Guimarães AG (2012). Carvacrol attenuates mechanical hypernociception and inflammatory response. Naunyn Schmiedebergs Arch. Pharmacol..

[76] Lee DK (2000). Characterization of apelin, the ligand for the APJ receptor. J. Neurochem..

[77] Park, C. S., Lee, J. Y., Choi, H. Y., Ju, B. G. & Yune, T. Y. Suppression of TRPM7 by carvacrol protects against injured spinal cord by inhibiting blood-spinal cord barrier disruption. (2021). 10.1089/neu.2021.0338 35171694

[78] Chodobski A, Zink BJ, Szmydynger-Chodobska J (2011). Blood–brain barrier pathophysiology in traumatic brain injury. Transl. Stroke Res..

[79] Lucas SM, Rothwell NJ, Gibson RM (2006). The role of inflammation in CNS injury and disease. Br. J. Pharmacol..

[80] Habib P, Dreymueller D, Ludwig A, Beyer C, Dang J (2013). Sex steroid hormone-mediated functional regulation of microglia-like BV-2 cells during hypoxia. J. Steroid Biochem. Mol. Biol..

[81] Westmoreland SV, Kolson D, Gonzalez-scarano F (1996). Toxicity of TNFα and platelet activating factor for human NT2N neurons: A tissue culture model for human immunodeficiency virus dementia. J. Neurovirol..

[82] Shohami E, Gallily R, Mechoulam R, Bass R, Ben-Hur T (1997). Cytokine production in the brain following closed head injury: Dexanabinol (HU-211) is a novel TNF-α inhibitor and an effective neuroprotectant. J. Neuroimmunol..

[83] Shojo H (2010). Genetic and histologic evidence implicates role of inflammation in traumatic brain injury-induced apoptosis in the rat cerebral cortex following moderate fluid percussion injury. Neuroscience.

[84] Bortolotti P, Faure E, Kipnis E (2018). Inflammasomes in tissue damages and immune disorders after trauma. Front. Immunol..

[85] Hoffmann A, Baltimore D (2006). Circuitry of nuclear factor κB signaling. Immunol. Rev..

[86] Lenardo MJ, Fan C-M, Maniatis T, Baltimore D (1989). The involvement of NF-κB in β-interferon gene regulation reveals its role as widely inducible mediator of signal transduction. Cell.

[87] Buhlman LM, Krishna G, Jones TB, Thomas TC (2021). Drosophila as a model to explore secondary injury cascades after traumatic brain injury. Biomed. Pharmacother..

[88] Caba E (2021). Excitotoxic stimulation activates distinct pathogenic and protective expression signatures in the hippocampus. J. Cell Mol. Med..

[89] Sivandzade F, Prasad S, Bhalerao A, Cucullo L (2019). NRF2 and NF-қB interplay in cerebrovascular and neurodegenerative disorders: Molecular mechanisms and possible therapeutic approaches. Redox Biol..

[90] Rickhag M (2006). Comprehensive regional and temporal gene expression profiling of the rat brain during the first 24 h after experimental stroke identifies dynamic ischemia-induced gene expression patterns, and reveals a biphasic activation of genes in surviving tissue. J. Neurochem..

[91] Sabetta Z, Krishna G, Curry T, Adelson PD, Thomas TC (2023). Aging with TBI vs. aging: 6-month temporal profiles for neuropathology and astrocyte activation converge in behaviorally relevant thalamocortical circuitry of male and female rats. bioRxiv.

[92] Thomas TC (2018). Does time heal all wounds? Experimental diffuse traumatic brain injury results in persisting histopathology in the thalamus. Behav. Brain Res..

[93] Bromberg CE (2020). Sex-dependent pathology in the HPA axis at a sub-acute period after experimental traumatic brain injury. Front. Neurol..

[94] Beitchman JA (2019). Experimental traumatic brain injury induces chronic glutamatergic dysfunction in amygdala circuitry known to regulate anxiety-like behavior. Front. Neurosci..

[95] Magierowicz K, Górska-Drabik E, Sempruch C (2019). The insecticidal activity of *Satureja*
*hortensis* essential oil and its active ingredient-carvacrol against *Acrobasis*
*advenella* (Zinck.) (Lepidoptera, Pyralidae). Pesticide Biochem. Physiol..

[96] Mazarei Z, Rafati H (2019). Nanoemulsification of *Satureja*
*khuzestanica* essential oil and pure carvacrol; comparison of physicochemical properties and antimicrobial activity against food pathogens. LWT.

[97] Freire CMM, Marques MOM, Costa M (2006). Effects of seasonal variation on the central nervous system activity of *Ocimum*
*gratissimum* L. essential oil. J. Ethnopharmacol..

[98] Soni U, Brar S, Gauttam VK (2015). Effect of seasonal variation on secondary metabolites of medicinal plants. Int. J. Pharm. Sci. Res..

[99] Najafian S, Zahedifar M (2015). Antioxidant activity and essential oil composition of *Satureja*
*hortensis* L. as influenced by sulfur fertilizer. J. Sci. Food Agric..

