# Supplementary material for: Satureja khuzistanica Jamzad essential oil and pure carvacrol attenuate TBI-induced inflammation and apoptosis via NF-κB and caspase-3 regulation in the male rat brain
Source: Sci Rep. 2023 Mar 23;13:4780. doi: 10.1038/s41598-023-31891-3 (PMC10036533; doi:10.1038/s41598-023-31891-3)
Supplement: Supplementary file 1 — Supplementary Legends. [file 41598_2023_31891_MOESM1_ESM.docx]

**Supplemental figure legends**

Supplemental figure 1. Schematic of Experimental Protocol. A total of 84 male Wistar rats were used in these experiments. One cohort was used for brain water content (n=6/group), another cohort was used for Western blot and ELISA (n=6/group), and a 3^rd^ cohort was used for histology (n=6/group).

Supplemental figure 2. Chromatogram report of SKEO where carvacrol is detected at 5.362’ and represents 91.33% of the essence.

Supplemental figure 3. H&E stained sections clearly demonstrate damage to the cortex after weight drop injury where a stainless steel disc (10mm diameter, 3mm depth) was attached to the skull midline between lambda and bregma using polyacrylamide.

Supplemental figure 4. Representative Western blots of (A) caspase:β-actin, (B) bax:β-actin, (C) bcl2:β-actin, and (D) NF-κB:β-actin. Yellow rectangles illustrate the location of the cropped bands used for the quantification of data for the figures in the manuscript.
