# Supplementary figures and images for: Satureja khuzistanica Jamzad essential oil and pure carvacrol attenuate TBI-induced inflammation and apoptosis via NF-κB and caspase-3 regulation in the male rat brain
Source: Sci Rep. 2023 Mar 23;13:4780. doi: 10.1038/s41598-023-31891-3 (PMC10036533; doi:10.1038/s41598-023-31891-3)

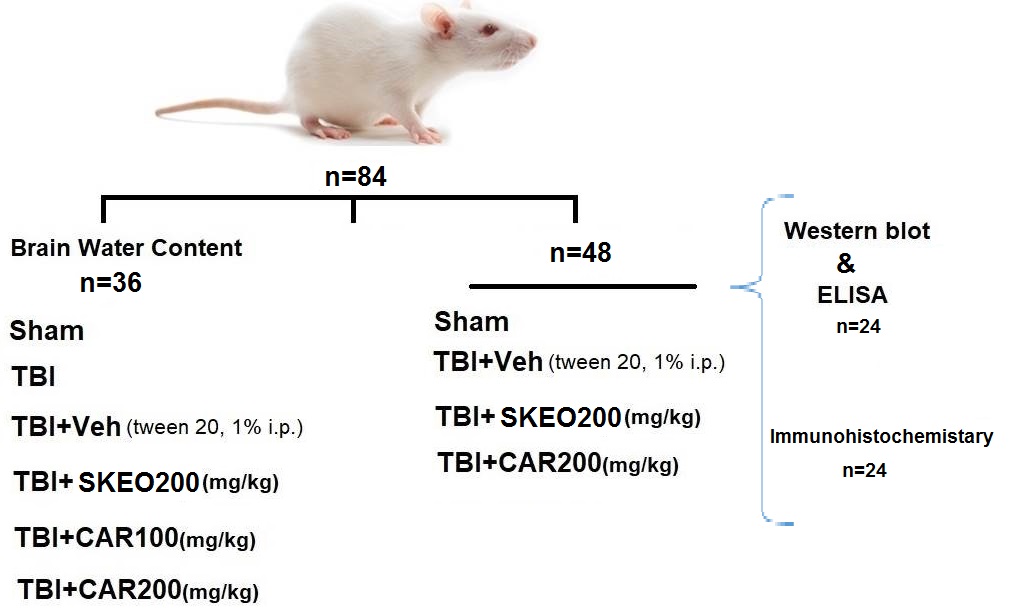

Supplement: Supplementary file 2 — Supplementary Figure 1. [file 41598_2023_31891_MOESM2_ESM.jpg]

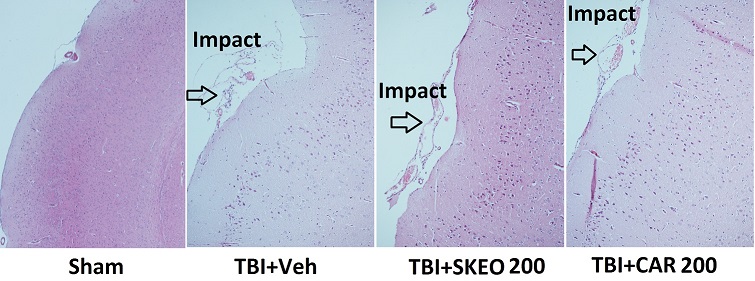

Supplement: Supplementary file 4 — Supplementary Figure 3. [file 41598_2023_31891_MOESM4_ESM.jpg]

A

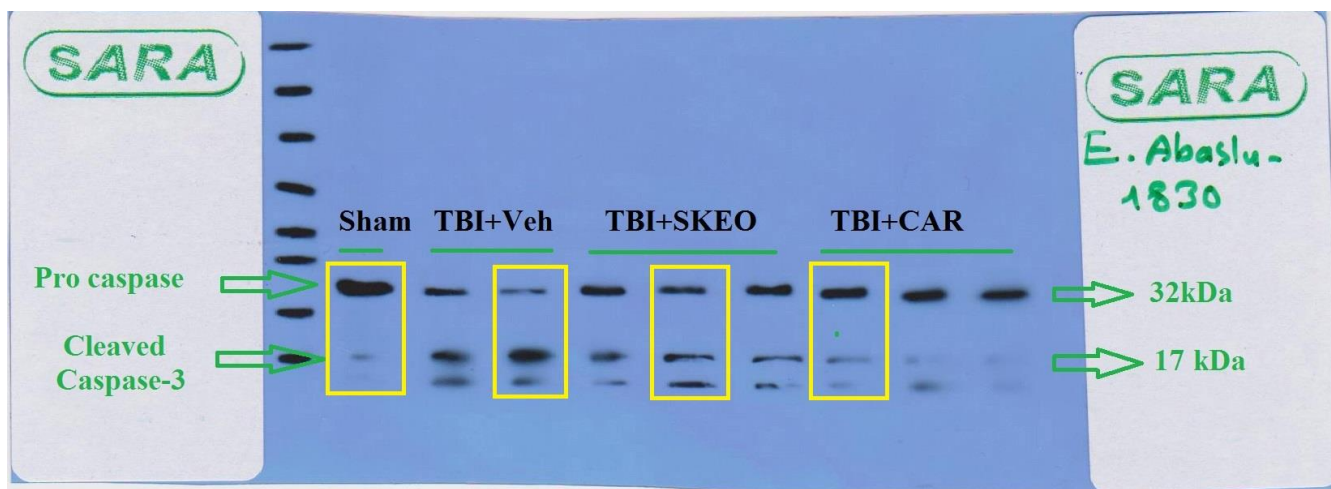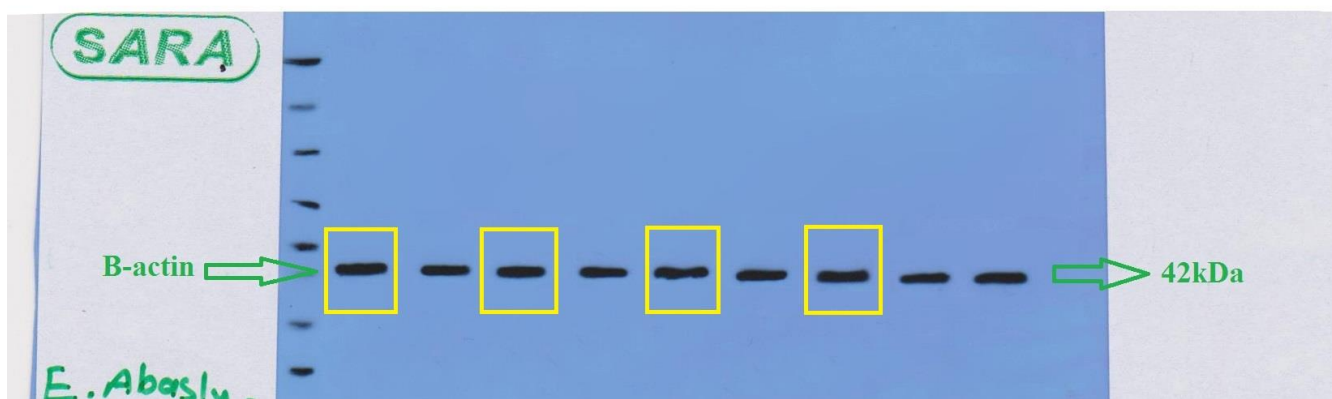

B

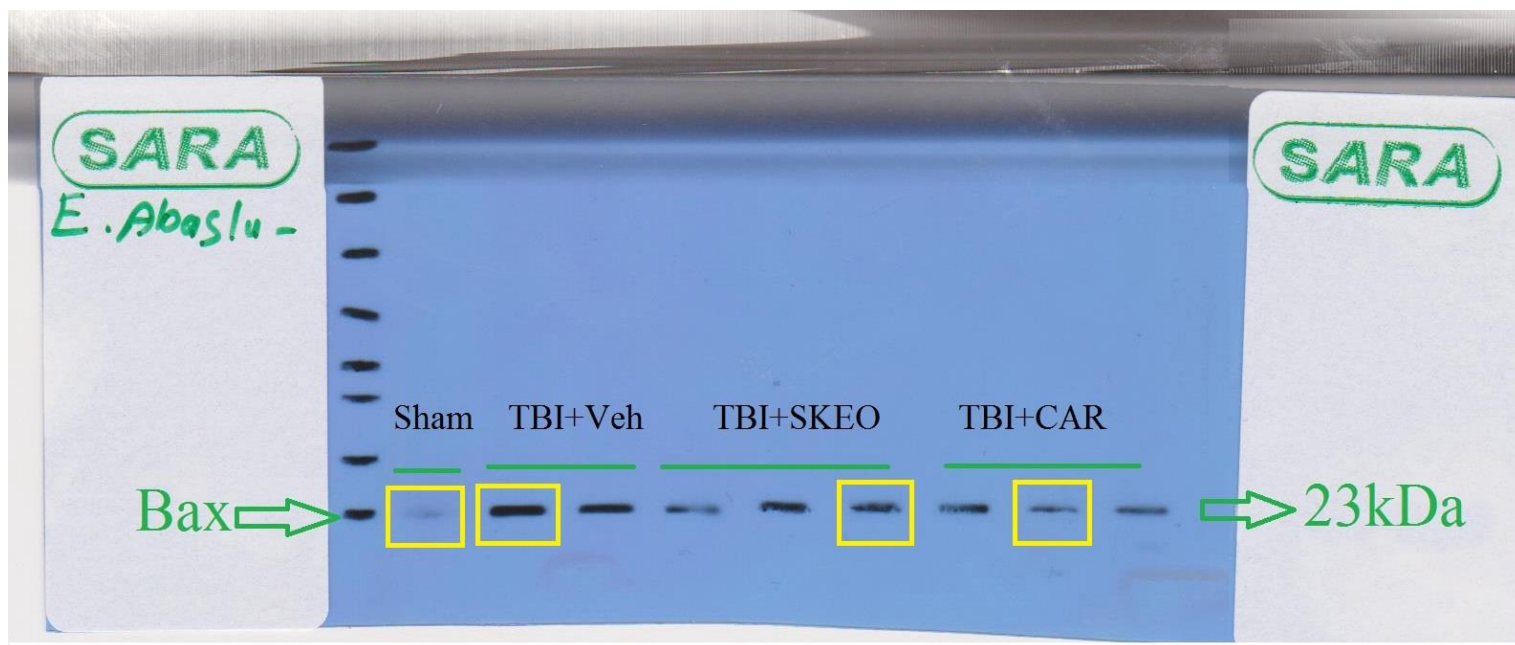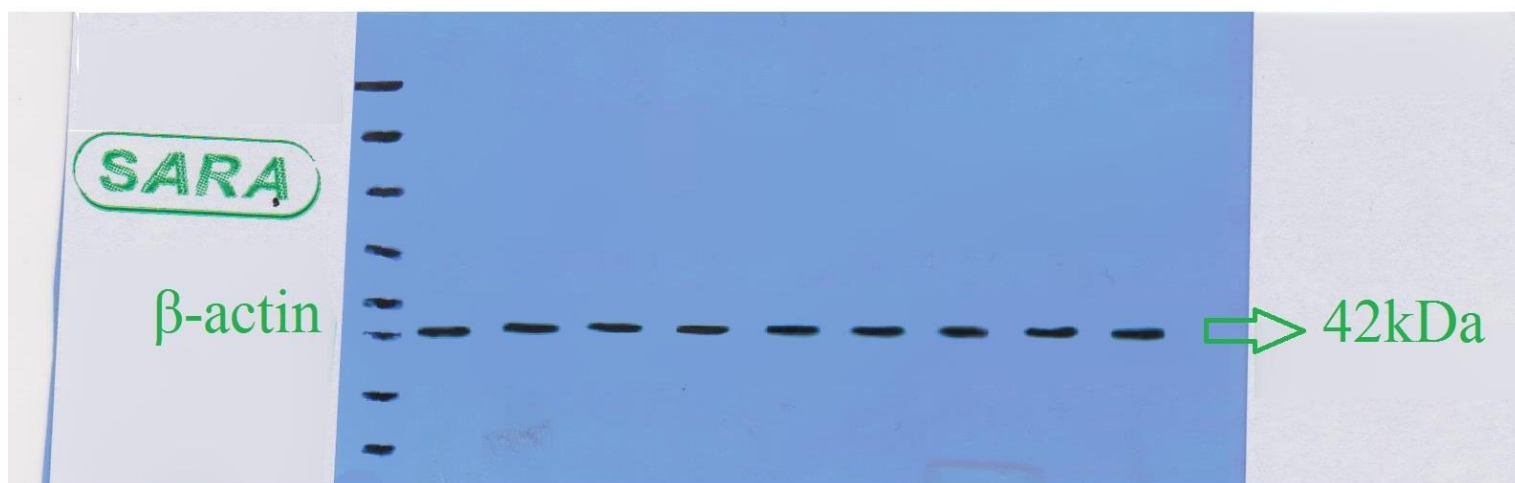

C

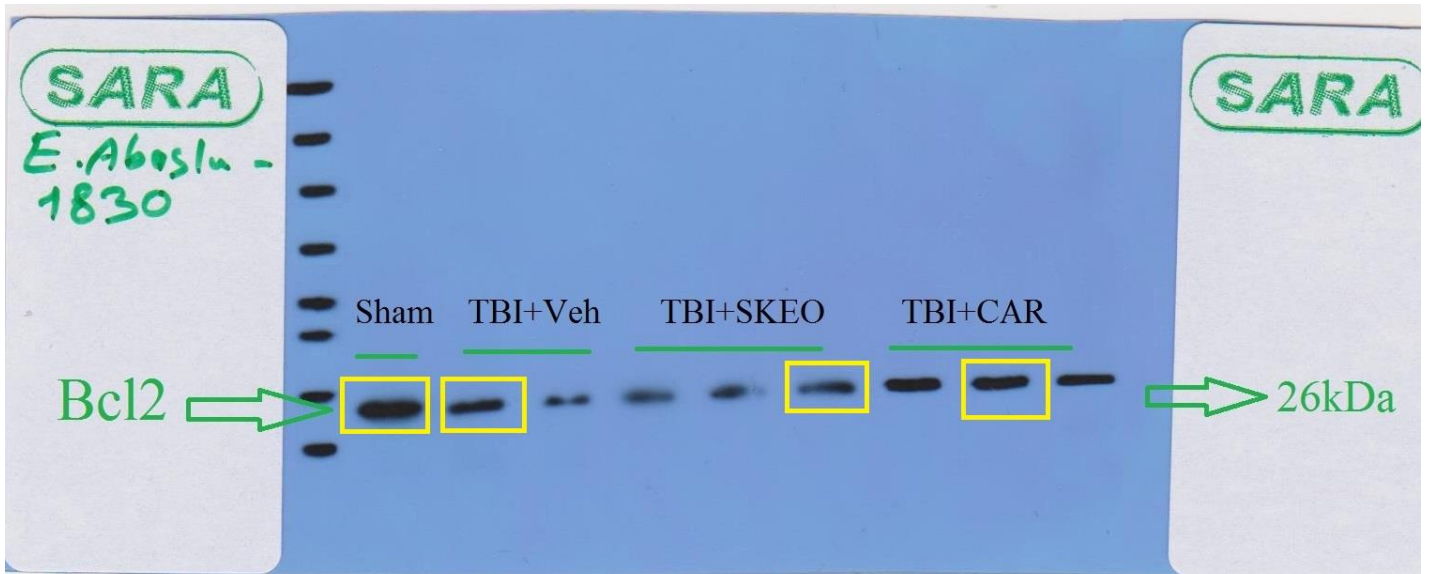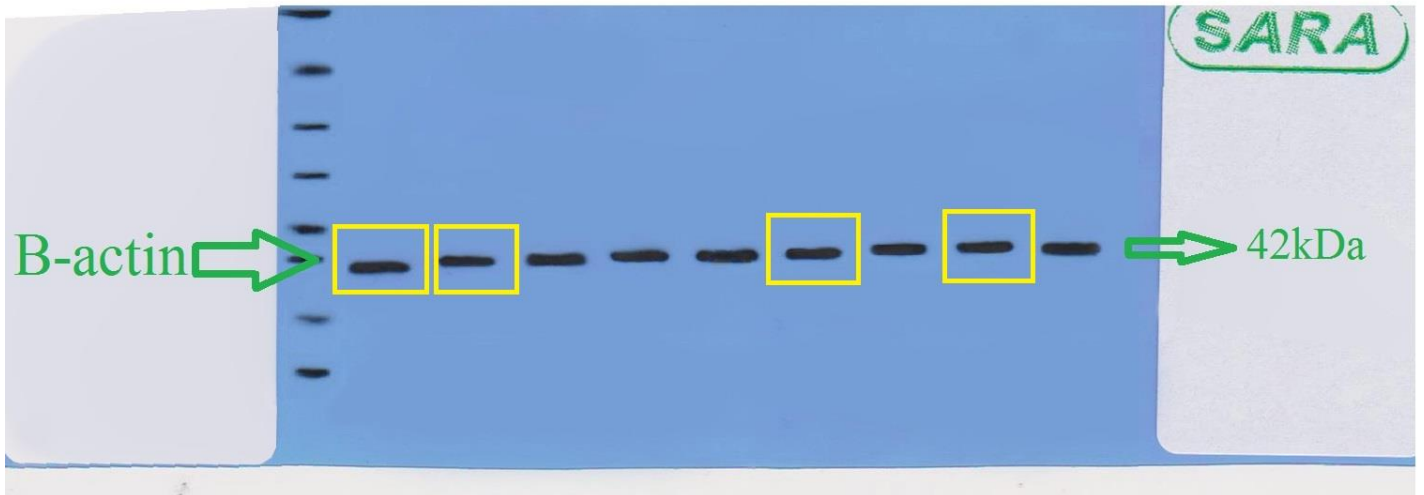

D

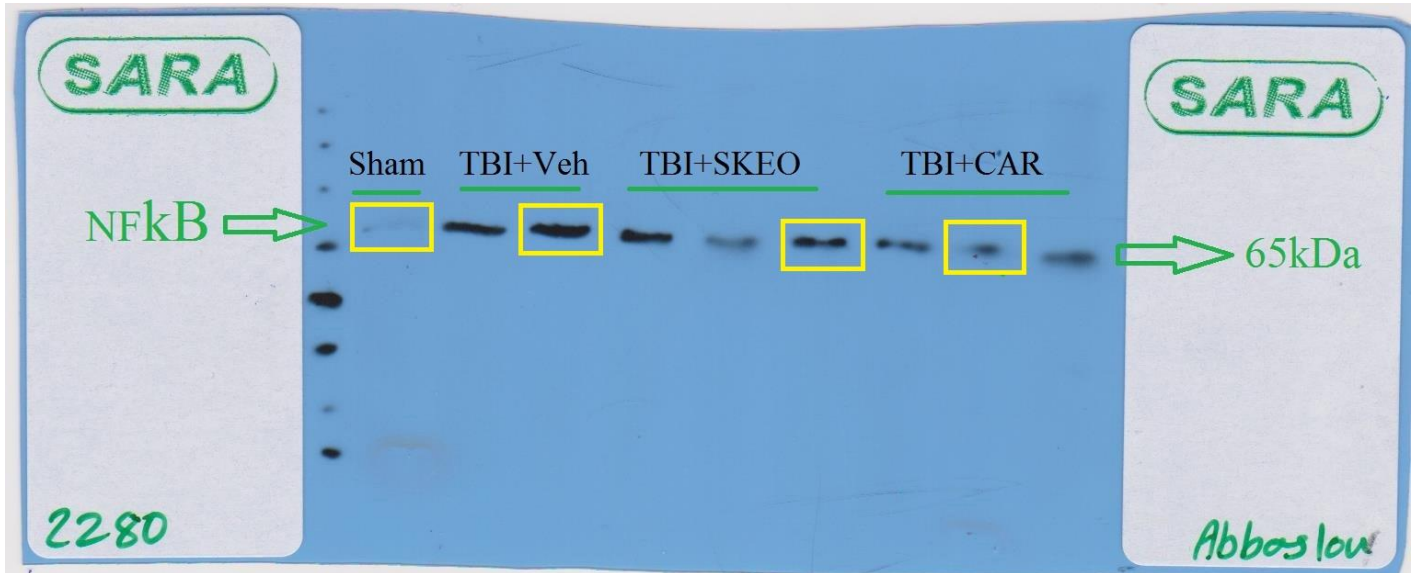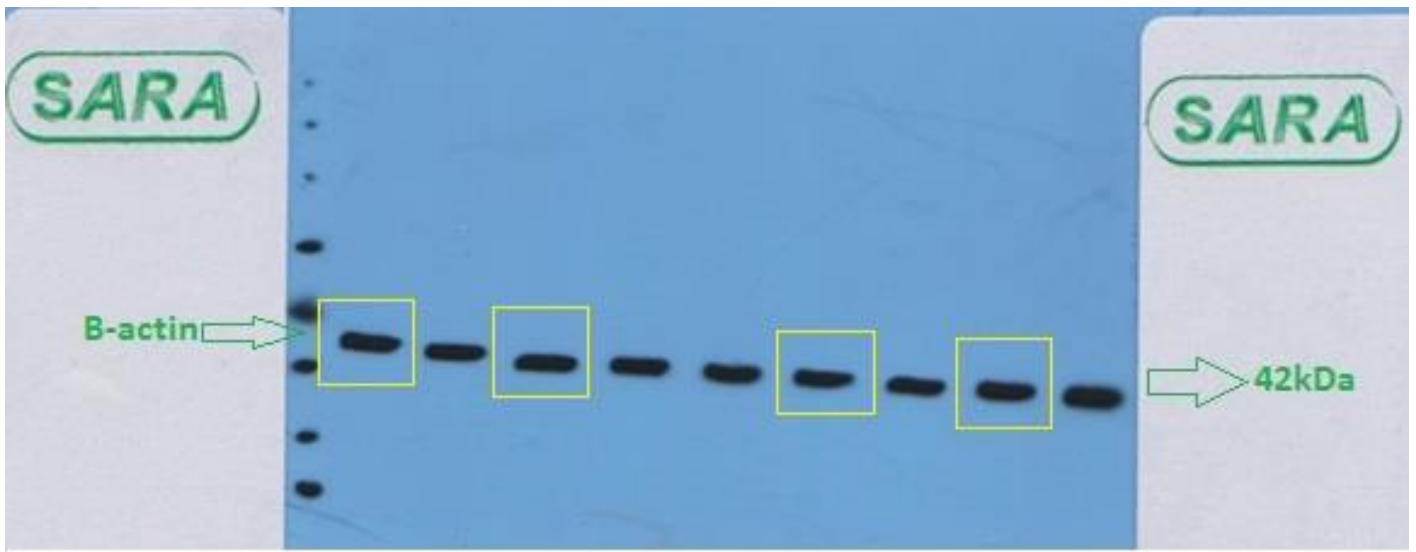

Supplement: Supplementary file 5 — Supplementary Figure 4. [file 41598_2023_31891_MOESM5_ESM.pdf]
