# Supplementary material for: Satureja khuzistanica Jamzad essential oil and pure carvacrol attenuate TBI-induced inflammation and apoptosis via NF-κB and caspase-3 regulation in the male rat brain
Source: Sci Rep. 2023 Mar 23;13:4780. doi: 10.1038/s41598-023-31891-3 (PMC10036533; doi:10.1038/s41598-023-31891-3)
Supplement: Supplementary file 3 — Supplementary Figure 2. [file 41598_2023_31891_MOESM3_ESM.pdf]

# Chromatogram Report

Essential oil *Satureja Khuzestanica* Jamzad: The first peak is related to the solvent and the second peak is related to Carvacrol.

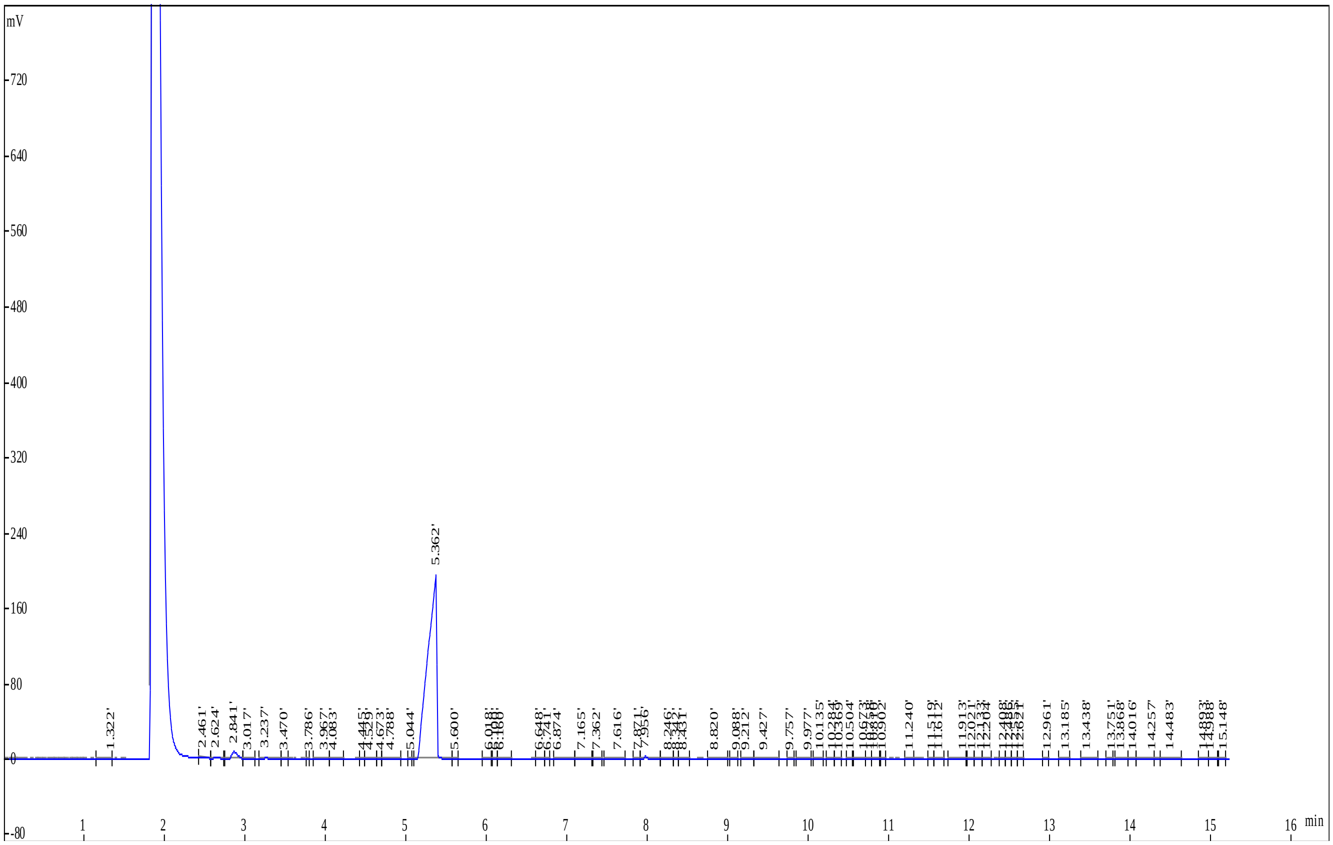

| Rank | Time | Name | Area% | Area |
|---|---|---|---|---|
| 1 | 1.322 | | 0.005525 | 88 |
| 2 | 2.461 | | 0.1169 | 1862 |
| 3 | 2.624 | | 0.3193 | 5088 |
| 4 | 2.841 | | 2.819 | 37984 |
| 5 | 3.017 | | 0.1097 | 1747 |
| 6 | 3.237 | | 0.8891 | 7237 |
| 7 | 3.470 | | 0.004029 | 64 |
| 8 | 3.786 | | 0.001657 | 26 |
| 9 | 3.967 | | 0.2517 | 4010 |
| 10 | 4.083 | | 0.06135 | 978 |
| 11 | 4.445 | | 0.002342 | 37 |
| 12 | 4.529 | | 0.05098 | 812 |
| 13 | 4.673 | | 0.00326 | 52 |
| 14 | 4.788 | | 0.03017 | 481 |
| 15 | 5.044 | | 0.02088 | 333 |
| 16 | 5.362 | | 91.3300 | 1511771 |
| 17 | 5.600 | | 0.03504 | 558 |
| 18 | 6.018 | | 0.008525 | 136 |
| 19 | 6.100 | | 0.02553 | 407 |

| | | | |
|---|---|---|---|
| 20 | 6.160 | 0.0386 | 615 |
| 21 | 6.648 | 0.005248 | 84 |
| 22 | 6.741 | 0.001751 | 28 |
| 23 | 6.874 | 0.1534 | 2445 |
| 24 | 7.165 | 0.03251 | 518 |
| 25 | 7.362 | 0.005762 | 92 |
| 26 | 7.616 | 0.01783 | 284 |
| 27 | 7.871 | 0.00828 | 132 |
| 28 | 7.956 | 0.6402 | 10203 |
| 29 | 8.246 | 0.008817 | 141 |
| 30 | 8.342 | 0.002606 | 42 |
| 31 | 8.431 | 0.05373 | 856 |
| 32 | 8.820 | 0.01343 | 214 |
| 33 | 9.088 | 0.002388 | 38 |
| 34 | 9.212 | 0.01191 | 190 |
| 35 | 9.427 | 0.04161 | 663 |
| 36 | 9.757 | 0.001536 | 24 |
| 37 | 9.977 | 0.004672 | 74 |
| 38 | 10.135 | 0.003425 | 55 |
| 39 | 10.284 | 0.00197 | 31 |
| 40 | 10.369 | 0.002993 | 48 |
| 41 | 10.504 | 0.001832 | 29 |
| 42 | 10.673 | 0.009226 | 147 |
| 43 | 10.758 | 0.003059 | 49 |
| 44 | 10.810 | 0.005095 | 81 |
| 45 | 10.902 | 0.002024 | 32 |
| 46 | 11.240 | 0.00545 | 87 |
| 47 | 11.519 | 0.001892 | 30 |
| 48 | 11.612 | 0.01244 | 198 |
| 49 | 11.913 | 0.008305 | 132 |
| 50 | 12.021 | 0.003262 | 52 |
| 51 | 12.133 | 0.00257 | 41 |
| 52 | 12.204 | 0.005301 | 84 |
| 53 | 12.408 | 0.002734 | 44 |
| 54 | 12.486 | 0.003467 | 55 |
| 55 | 12.555 | 0.00444 | 71 |
| 56 | 12.621 | 0.00705 | 112 |
| 57 | 12.961 | 0.002578 | 41 |
| 58 | 13.185 | 0.01608 | 256 |
| 59 | 13.438 | 0.0138 | 220 |
| 60 | 13.751 | 0.002121 | 34 |
| 61 | 13.868 | 0.01039 | 166 |
| 62 | 14.016 | 0.01005 | 160 |
| 63 | 14.257 | 0.01025 | 163 |
| 64 | 14.483 | 0.04716 | 752 |
| 65 | 14.893 | 0.005143 | 82 |
| 66 | 14.988 | 0.002723 | 43 |
| 67 | 15.148 | 0.001544 | 25 |
| Total | | 100 | 1593634 |
